# Turbulence drive and causal generation of vorticity in edge fusion plasmas

**DOI:** 10.1007/s41614-025-00197-4

**Published:** 2025-07-08

**Authors:** G. Dif-Pradalier, Y. Sarazin, Ph. Ghendrih, F. Widmer, Y. Camenen, P. Donnel, X. Garbet, V. Grandgirard, A. Jamann, K. Obrejan, M. Protais, R. Varennes

**Affiliations:** 1 CEA, IRFM, F-13108 Saint-Paul-lez-Durance, France; 2 Max Planck Institute for Plasma Physics, D-85748 Garching, Germany; 3 Aix-Marseille Université, CNRS PIIM, UMR 7345, Marseille, France; 4 School of Physical and Mathematical Sciences, Nanyang Technological University, Singapore, 637371 Singapore

**Keywords:** Turbulence, plasmas, confinement, shortfall, spreading, causality

## Abstract

The edge of fusion plasmas is particularly important to model as this is where the plasma self-organizes to higher-energy states. From a turbulence point of view, it is a difficult region to model, often marginally stable, possibly deviating from a quasilinear approximation and subject to the influence of the material boundaries. A "transport shortfall", i.e., an underprediction of turbulence transport, has often been reported at the plasma edge. The integration of core, edge and scrape-off layer (SOL) dynamics is an area of active investigation. We have recently provided evidence for an active interplay between the confined plasma and its material boundaries. This interplay (i) cures the 'transport shortfall' at the edge of the plasma column and leads to (ii) the onset of a spontaneous, albeit moderate, transport barrier at the plasma edge, the mechanisms of which have been studied in detail. The present paper is the companion manuscript to [Dif-Pradalier, G., et al. Commun Phys 5, 229 (2022) Dif-Pradalier et al. (2022)]. We provide here comprehensive details of the techniques and procedures used in the earlier paper, including the detailed linear stability of the plasma edge, nonlinear analysis for edge turbulence, the importance of forcing and boundary conditions, and further details of the method by which causality was assessed. It also highlights the central role of diamagnetic flows in the generation of the edge transport barrier.

## Introduction

The peripheral edge of low confinement mode plasmas (L mode) poses significant modeling challenges, yet it is crucial to fusion as turbulent fluctuations can organize into self-generated flows, transport barriers and lead to potentially drastic changes in confinement. Experimental measurements of the relative turbulence fluctuation levels $\delta n/n$, where *n* is the plasma density, almost invariably show a robust increase from the core to the magnetic separatrix (Liewer 1985). However, the origin and the nature of these turbulent fluctuations at the plasma edge are often unclear and subject to speculation. Linear analysis of the underlying profiles can result in the plasma edge being classified as either linearly unstable, marginally unstable, or even, often, linearly stable. This creates a significant point of contention for models as transport predictions when approaching the separatrix can vary considerably when modest modifications are made to the plasma profiles, as demonstrated in Ref. Görler et al. (2014).

This result demonstrates a propensity for "stiffness" in the edge profiles. When profiles are stiff, minor alterations in profile gradients can result in substantial fluctuations in fluxes. This variability is unsatisfactory in that it lacks the robustness of the experimental observations of relative turbulence fluctuation levels, which increase from core to separatrix. Furthermore, it sheds important doubts on the ability of the models to accurately capture the complex nonlinear dynamics at play. This variability (or stiffness), however, is commonplace near marginal stability (Diamond and Hahm 1995; Dif-Pradalier et al. 2017; Peeters et al. 2016; Gillot et al. 2023) or in the plasma edge although possibly not universal (Sauter et al. 2014). Constraining profiles or gradients can provide an *interpretative* explanation of certain observations, but the variability that accompanies ‘stiffness’ casts doubt on the *predictive* capabilities of such approaches to capture basic mechanisms when these are intrinsically nonlinear in nature. Rather, one should impose sources that are independent of the dynamics and have fluxes, stresses, *along with the profiles themselves*, as a result of the nonlinear organization. The latter approach is often termed ‘flux-driven’ in contrast to the former, which is termed ‘gradient-driven’.

One of the objectives of Ref. Dif-Pradalier et al. (2022) and of the present paper, as its companion, is to illustrate a generic means by which, irrespective of the precise underlying gradients, nonlinear mechanisms can robustly provide a means to recover experimentally-relevant levels of turbulence activity in the plasma edge. Given that edge turbulence activity controls stress and electric field dynamics and affects access to bifurcated transport states or deposition patterns on plasma-facing components, understanding the origin and magnitude of edge turbulence is highly relevant to fusion.

An important aspect of the work is to highlight the close interplay between the confined, dense and hot core of fusion plasmas, the unconfined peripheral boundary layer (the ’scrape-off layer’ or ’SOL’) and the loosely defined ’edge’ region in between. This has been speculated for years: edge turbulence may be partly or wholly due to ‘spreading’ of fluctuations (Garbet et al. 1994) from the core (Mattor and Diamond 1994; Hahm et al. 2004; Gürcan et al. 2013) or from the SOL (Kadomtsev and Laing 1993).

We have recently provided evidence in support of these ideas, highlighting two key elements (Dif-Pradalier et al. 2022): (i) turbulent fluctuations observed at the edge have originated elsewhere and have expanded beyond their region of linear instability drive. The actual spreading of fluctuations is mainly from the separatrix region and inwards, initially. In steady state however, spreading of core turbulence into the edge is important to sustain high fluctuation levels there. However, before fluctuations can spread, they must be born; this is where (ii) the plasma boundary interplay plays a critical role, or more generally, where the introduction of a localized cold sink in a small region of the plasma edge deeply affects the nonlinear dynamics, globally. This localized cold region can be a limiter or perhaps even a radiative X-point. Importantly, it will eventually lead to free-energy injection, i.e., further destabilization of the plasma edge, far beyond the possibly small region from which it originated.

These two elements have interesting implications. Although the edge is marginally unstable or even linearly stable, the experimental trend of relative fluctuation levels, which is observed to increase from the core to the separatrix, is robustly recovered, quantitatively. We have also reported there the spontaneous generation of a sheared poloidal flow. The present paper presents additional nonlinear evidence for the above, discusses the linear stability of the edge plasmas, the importance of forcing and boundary conditions and the method by which causality was assessed to understand shear flow onset at the plasma edge.

## Model equations

Low-frequency microturbulence in weakly collisional magnetized plasmas is appropriately described within the gyrokinetic framework (Brizard and Hahm 2007). The gysela code (Grandgirard et al. 2016) solves the governing coupled gyrokinetic, quasi-neutrality and Ampère equations for the full guiding-center distribution function ${\bar{F}}_s$ of species *s* (ions or electrons), in five-dimensional guiding-center space $({\textbf{x}}_G,v_{G\Vert },\mu )$ and time. Fully kinetic, hybrid kinetic or adiabatic (Boltzmann) electron models are available (Donnel et al. 2025) to solve quasineutrality and the mixed variable scheme (Mishchenko et al. 2017; Gillot 2020) is used to solve the Ampére equation, reducing numerical inaccuracies common in electromagnetic gyrokinetic simulations. In the present work, we restrict ourselves to a subset of these capabilities: we consider an electrostatic framework and a simplified adiabatic electron response, which assumes that electrons are always at equilibrium with electrostatic potential fluctuations. The relevant equations read:

1 $$\begin{aligned}&B_{\Vert s}^*\frac{\partial {{\bar{F}}_s}}{\partial {t}} + {\pmb \nabla }\cdot \left( B_{\Vert s}^*\frac{\textrm{d}{\textbf{x}}_G}{\textrm{d}t} {\bar{F}}_s\right) + \frac{\partial {\,}}{\partial {v_{G\Vert }}}\left( B_{\Vert s}^*\frac{\textrm{d}v_{G\Vert }}{\textrm{d}t} {\bar{F}}_s\right) = B_{\Vert s}^*\left[ \text {rhs}\right] \end{aligned}$$

2 $$\begin{aligned}&\text {rhs}= {\mathcal {C}} + {\mathcal {S}} + {\mathcal {D}} - \nu \left[ {\mathcal {M}}^{\mathfrak {mat}}(r,\theta )+ {\mathcal {M}}^\mathrm{{uSOL}}(r)\right] [{\bar{F}}_s- {\mathbb {G}}_{\mathrm{{cold}}}] -\,\gamma ^{_K} \nonumber \\&\quad \left[ {\bar{F}}_s- {\langle {\text {f}}\rangle }_{\text {F-D}} \left( 1 + \dfrac{ \langle {\bar{F}}_s- {\langle {\text {f}}\rangle }_{\text {F-D}} \rangle }{ \langle {\langle {\text {f}}\rangle }_{\text {F-D}} \rangle } \right) \right] \end{aligned}$$

3 $$\begin{aligned}&\frac{T_e}{e} \bigg [\rho + \frac{1}{n_{e_0}} \sum _s Z_s\nabla _\perp \cdot \left( \frac{n_{s_0}}{B\Omega _s}\nabla _\perp \phi \right) \bigg ] = \phi - \lambda \Big [ 1-{\mathcal {M}}^\mathrm{{SOL}}(r)\Big ] \langle \,{\phi }\,\rangle _{\textrm{FS}} \nonumber \\&\qquad - \Big [{\mathcal {M}}^{\mathfrak {mat}}(r,\theta )-{\mathcal {M}}^\mathrm{{wall}}(r)\Big ] \phi ^{\text {bias}} - \lambda \Lambda \Big [ {\mathcal {M}}^\mathrm{{SOL}}(r)-{\mathcal {M}}^{\mathfrak {mat}}(r,\theta )\Big ] (T_e - T_e^\mathrm{{b.c.}}) \end{aligned}$$

4 $$\begin{aligned}&\rho ({\textbf{x}},t) = \frac{1}{n_{e_0}}\sum _s Z_s\int \,\textrm{d}\mu {\mathcal {J}}_\mu .\left[ \int J_{\textrm{v}}\,\textrm{d}v_{G\Vert }\; ({\bar{F}}_s({\textbf{x}},{\textbf{v}},t)-{\bar{F}}_{s,\textrm{eq}}(r,\theta ,v_{G\Vert }))\right] \end{aligned}$$

with $J_{\textrm{v}}$ and $J_{\textrm{x}}$ being the velocity and the space Jacobians, ${\mathcal {J}}_\mu .$ the gyro-average operator, $\rho$ the charge density of guiding-centers and $\langle \cdot \rangle =\int J_{\textrm{v}}J_{\textrm{x}}\, \cdot \,\textrm{d}v_\parallel \textrm{d}\mu \textrm{d}\theta \textrm{d}\varphi$. Notations are those of Ref. Grandgirard et al. (2016). The computational domain extends from inner core ($r/a=0$) to the material boundaries (typically, about $r/a=1.3$). The polarization density in Eq.(3) is written in the long wavelength approximation, which is appropriate for adiabatic calculations of the Ion Temperature Gradient instability (Idomura 2012). When considering a kinetic response for the electrons and in the presence of large poloidal asymmetries (as in the case of a limiter), the more accurate Padé approximation of the polarization density, recently implemented in gysela (Donnel et al. 2025), may become important. Finally, the adiabatic assumption postulated in Eq.(3) implies a subdominant contribution of convective particle transport. The present model retains merit up to the separatrix; however, the lack of convective transport precludes the drawing of meaningful conclusions about the dynamics of the scrape-off layer.

Flux- or gradient-driven dynamics are considered. A detailed discussion of the physical implications of flux-versus-gradient-driven descriptions is forthcoming in Ref. Dif-Pradalier et al. (2025). For flux-driven evolution, $\gamma ^{_K}=0$ in Eq.(2) and the distribution function evolves according to volumetric sources ${\mathcal {S}}$ or ${\mathcal {D}}$ (Sarazin et al. 2011) and penalized (Paredes et al. 2014; Caschera et al. 2018; Dif-Pradalier et al. 2022) heat and momentum sinks: ${\mathcal {M}}^\mathrm{{uSOL}}(r)$ for Case 2 and ${\mathcal {M}}^{\mathfrak {mat}}(r,\theta )$, ${\mathcal {M}}^\mathrm{{SOL}}(r)$ and ${\mathcal {M}}^\mathrm{{wall}}(r)$ for Case 1. Both these series of masks ${\mathcal {M}}^\mathrm{{uSOL}}(r)$, ${\mathcal {M}}^{\mathfrak {mat}}(r,\theta )$, ${\mathcal {M}}^\mathrm{{SOL}}(r)$ and ${\mathcal {M}}^\mathrm{{wall}}(r)$, combinations of hyperbolic tangents and the sources ${\mathcal {S}}$ or ${\mathcal {D}}$ are easily adjustable in location, shape and stiffness, providing great flexibility in mimicking various geometries or heating/cooling conditions. For gradient-driven evolution, ${\mathcal {S}}={\mathcal {D}}={\mathcal {M}}^\mathrm{{uSOL}}(r)={\mathcal {M}}^{\mathfrak {mat}}(r,\theta )={\mathcal {M}}^\mathrm{{SOL}}(r)={\mathcal {M}}^\mathrm{{wall}}(r)=0$ and $\gamma ^{_K} \ne 0$. Four cases are considered here (Cases 1, 2 and 3 are the same as in Dif-Pradalier et al. (2022), Case-tb below is only discussed here):

**Fig. 1:**
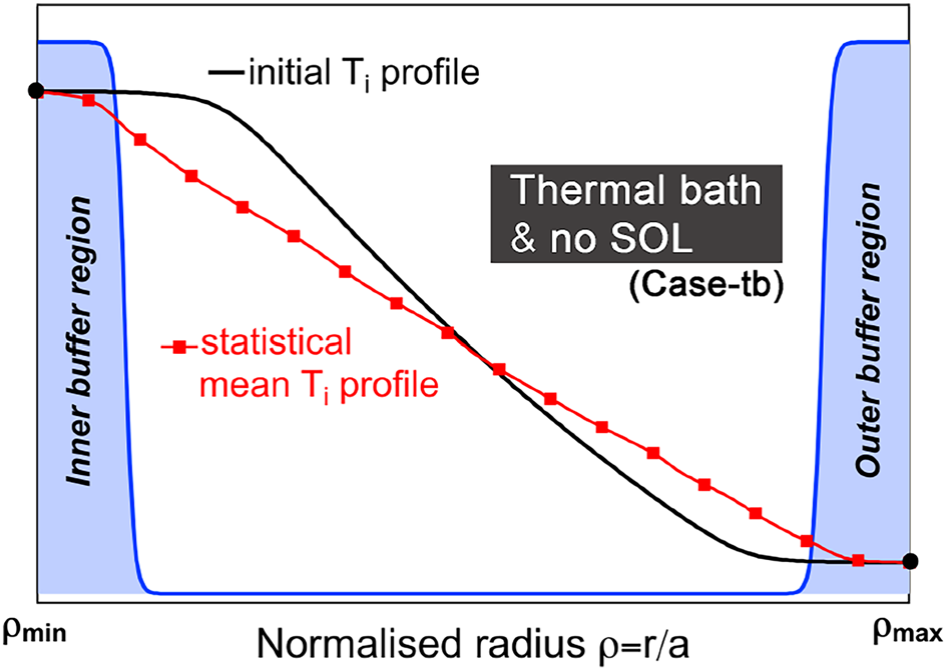
Schematic view of the “thermal bath" forcing (Case tb), shown here on the ion temperature. Flux coupling with the boundaries is allowed through non-vanishing boundary gradients at $\rho _{\text {min}}$ and $\rho _{\text {max}}$. The system is flux-driven. These boundary conditions however couple (extract) an undisclosed and somewhat artificial amount of free energy to (from) the system (Dif-Pradalier et al. 2025)

1. “**limiter and SOL**", is **flux-driven**. It allows description of the closed (core and edge) to open field line (SOL) transition through description of how the magnetic field lines from the hot plasma directly impinge on a toroidal material surface called a ‘limiter’. This configuration has been used for years in tokamak devices and is still used either in ramp-up phases of most current and future devices or as a means to protect sensitive components such as antennas. It has ${\mathcal {S}}\ne 0$ and ${\mathcal {D}}={\mathcal {M}}^\mathrm{{uSOL}}(r)=0$. Various shapes of limiters can be explored in gysela while specifying the precise shape of ${\mathcal {M}}^{\mathfrak {mat}}(r,\theta )$. We have not found significant differences, should the limiter be flat or domed. In the following, the limiter considered is toroidally symmetric and flat. The vertical ${\textbf{B}}\times \nabla B$ magnetic drift of the ions points toward it. The point of contact with the plasma is at $(r/a=1;\ \theta =-90^\circ )$, the poloidal angle $\theta$ is expressed in degrees. How the poloidal location of the limiter affects the results will be reported elsewhere. The current work uses the geometry detailed in Ref. Dif-Pradalier et al. (2022), Fig. 6a for ${\mathcal {M}}^\mathrm{{SOL}}(r)$, Fig. 6b for ${\mathcal {M}}^\mathrm{{wall}}(r)$ and Fig. 6d for ${\mathcal {M}}^{\mathfrak {mat}}(r,\theta )$. Finally, we cannot currently model an X-point geometry within gysela and thus address divertor configurations. The mechanisms advocated below, which occur when the plasma is in contact with a cold localized region, seem generic enough that the physics inferred here for a limiter could be extended to radiative X-point scenarios;
2. “**uniform SOL**", is **flux-driven**. Contrary to Case-1, no material boundary is modeled here and transition for the closed to open field lines is crudely modeled through application of a poloidally uniform mask ${\mathcal {M}}^\mathrm{{uSOL}}(r)$, where relaxation toward a cold Maxwellian is enforced, through a BGK operator. Here, ${\mathcal {S}}\ne 0$ and ${\mathcal {D}}={\mathcal {M}}^{\mathfrak {mat}}(r,\theta )={\mathcal {M}}^\mathrm{{SOL}}(r)={\mathcal {M}}^\mathrm{{wall}}(r)=0$. The shape of ${\mathcal {M}}^\mathrm{{uSOL}}(r)$ resembles that of ${\mathcal {M}}^\mathrm{{SOL}}(r)$ in Ref. Dif-Pradalier et al. (2022), Fig. 6a. Case-2 crudely mimics poloidally uniform boundary conditions, with transition to a uniform scrape-off layer. It is flux-driven and as such self-consistently includes turbulence spreading with respect to the gradient-driven approach, Case 3 below, but without the added physics of plasma-material boundary interactions described in Case 1;
3. “**gradient-driven and SOL**", is **gradient-driven**. In order to precisely compare apples to apples, the target distribution function ${\langle {\text {f}}\rangle }_{\text {F-D}}$, which is the base state about which the dynamics is forced to unfold, is chosen to be the statistical distribution at equilibrium from flux-driven Case 2. The relaxation rate $\gamma ^{_K} = 5.43\,10^{-5} \sim \gamma _{lin}/10$ is an order of magnitude smaller than the local linear turbulence growth rate $\gamma _{lin}$ at $r/a=0.7$. The BGK operator [last term of Eq.(2)] which allows to mimic a gradient-driven framework within gysela is specifically described in Ref. Dif-Pradalier et al. (2017) and built such as to prevent overdamping zonal modes (McMillan et al. 2008). Importantly, the gradient-driven approach has been shown to damp out zonal mean flows (Dif-Pradalier et al. 2017; Peeters et al. 2016), hinder turbulence spreading (Dif-Pradalier et al. 2022) and staircase formation (Peeters et al. 2016; Dif-Pradalier et al. 2017) as compared to the flux-driven frameworks which do not postulate scale separations (Dif-Pradalier et al. 2025);
4. “**thermal bath and no SOL**", is **flux-driven**. The setup is similar to Case 2 except for two aspects: (i) no SOL is considered here, and (ii) the system is supplemented with buffer boundary regions which replace the crude SOL regions of Case 2, as shown in Fig. 1, where diffusion ${\mathcal {D}}( {\bar{F}}_s)=\partial _r \left[ r\, {\mathfrak {D}}\, \partial _r \big \{ {\bar{F}}_s-f_{s,\text {target}} \big \} \right] /r$ is artificially introduced. Neither a volumetric source ${\mathcal {S}}=0$ nor masks ${\mathcal {M}}^\mathrm{{uSOL}}(r)={\mathcal {M}}^{\mathfrak {mat}}(r,\theta )={\mathcal {M}}^\mathrm{{SOL}}(r)={\mathcal {M}}^\mathrm{{wall}}(r)=0$ are considered. Diffusion ${\mathfrak {D}}>0$ is typically large in the buffer regions (with respect to effective diffusion elsewhere from the turbulence) so as to ensure both numerical stability (damping of turbulent fluctuations) and effective flux coupling to the external thermal baths. Here, $f_{s,\text {target}}$ is chosen as the local Maxwellian at both radial boundaries which has the initial density and temperature of ${\bar{F}}_s(t=0)$. The region of computational interest lies between the inner and outer buffer regions. There, mean profiles and fluctuations evolve freely, subject to the fluxes that cross the buffer boundaries.

Penalization of the quasineutrality equation (3) only applies to Case 1 and is such that, in the SOL, the electric potential $\phi$ relaxes toward its expected pre-sheath condition $\Lambda T_e/e$. Additionally, $\phi$ may be biased to $\phi ^{\text {bias}}$ in the limiter ($\phi ^{\text {bias}}=0$ in the current study) and is freely evolving elsewhere. $T_{e}^\mathrm{{b.c.}}$ is the cold electron temperature of the limiter and wall, chosen as the minimum $T_e$ value within the computational domain, $\Lambda =\log (\sqrt{m_i/m_e})$ and coefficient $\lambda$ (set to unity in the present study) may be used to alter the inertia of the zonal potential. In the gyrokinetic equation, infinite penalization (Caschera et al. 2018) relaxes ${\bar{F}}_s$ to a target cold Maxwellian distribution function ${\mathbb {G}}_{\mathrm{{cold}}}= n_{\text {w}} (2\pi T_{\text {w}})^{-3/2}\exp [-(v_{G\Vert }^2+\mu B)/ 2 T_{\text {w}}]$, characterized by low wall thermal energy $T_{\text {w}}$ and target density $n_{\text {w}}$. The former is constrained by velocity–space resolution. We typically choose it an order of magnitude lower than temperature at mid radius, while the target density $n_{\text {w}}$ is chosen so as to ensure particle conservation.

## Turbulent (nonlinear) organization of the plasma edge

**Fig. 2:**
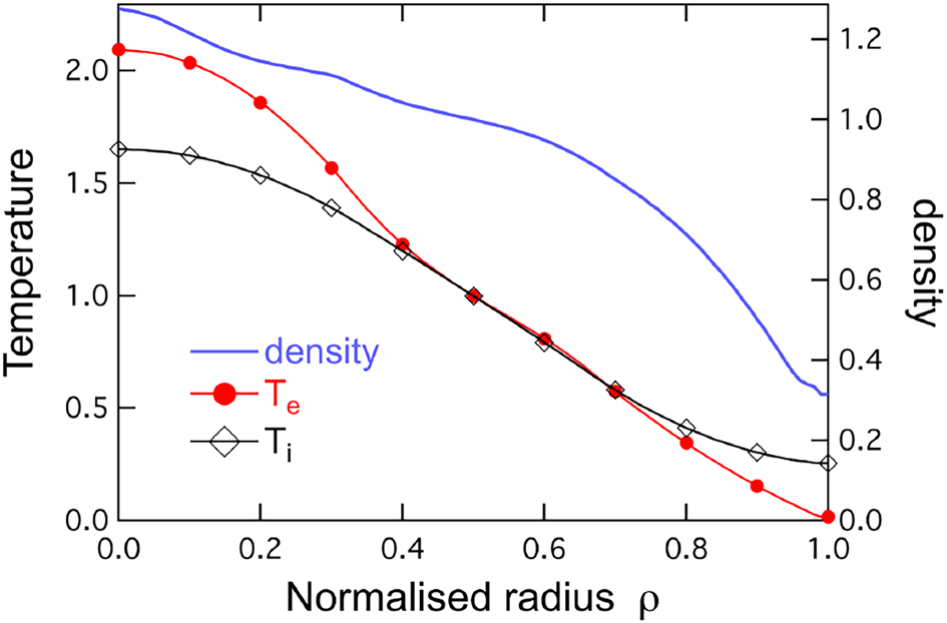
Initial density, electron and ion temperature profiles used for all computations, taken from the Tore Supra shot 45511. Density and $T_e$ profiles are fixed; the ion temperature profile $T_i$ varies in a flux-driven context, is fixed in a gradient-driven framework

All parameters disclosed in the paper are based on those of the Tore Supra shot 45511. In this experiment, a deuterium plasma of normalized size $\rho _\star =\rho _i/a =1/500$ at mid radius and aspect ratio $a/R_0=1/3.3$ is heated by 2MW of Ion Cyclotron Resonance Heating and 1MW of Ohmic heating. Here, $\rho _i$ is the ion Larmor radius, *a* and $R_0$ the minor and major radii, respectively. A 0.8MA current runs through the plasma, confined by a $B_0=2.8$T magnetic field on axis. Mid-radius density *n* and temperature *T* are respectively: $n=4\,10^{19}$ $\hbox {m}^{-3}$ and $T= 0.8$keV.

In gysela, a 3MW volumetric heat source comparable in shape to that in the experiment is injected in the central 40% of a deuterium torus of same aspect ratio. Initial density, electron and ion temperature profiles, shown in Fig. 2, are the same as in the experiment up to the separatrix. In the core $T_e/T_i > 1$, this ratio reverses in the edge and SOL, as in the experiment. To offset the numerical cost of the computations, a reduced magnetic field on axis $B_0=1.7$T is assumed, which amounts to computing a plasma column of smaller size $\rho _\star =1/300$ on a 1/4 wedge torus and $(r,\theta ,\varphi , v_\parallel ,\mu ) = (512,1024,64,128,64)$ grid.

### Without modeling the scrape-off layer (SOL)

We test the premise that it is sufficient to model the confined core and edge to compute the way turbulence organizes in the plasma edge. The hypothesis is first tested in the flux-driven framework of the "thermal bath" forcing (Case tb), which is somewhat restrictive. The objective is to assess the feasibility of modeling turbulence levels and subsequent turbulence organization in the plasma edge based solely on experimental profiles, without delving into the specifics of boundary conditions and proper forcing. The results, as illustrated below, indicate that this approach would not be viable.

**Fig. 3:**
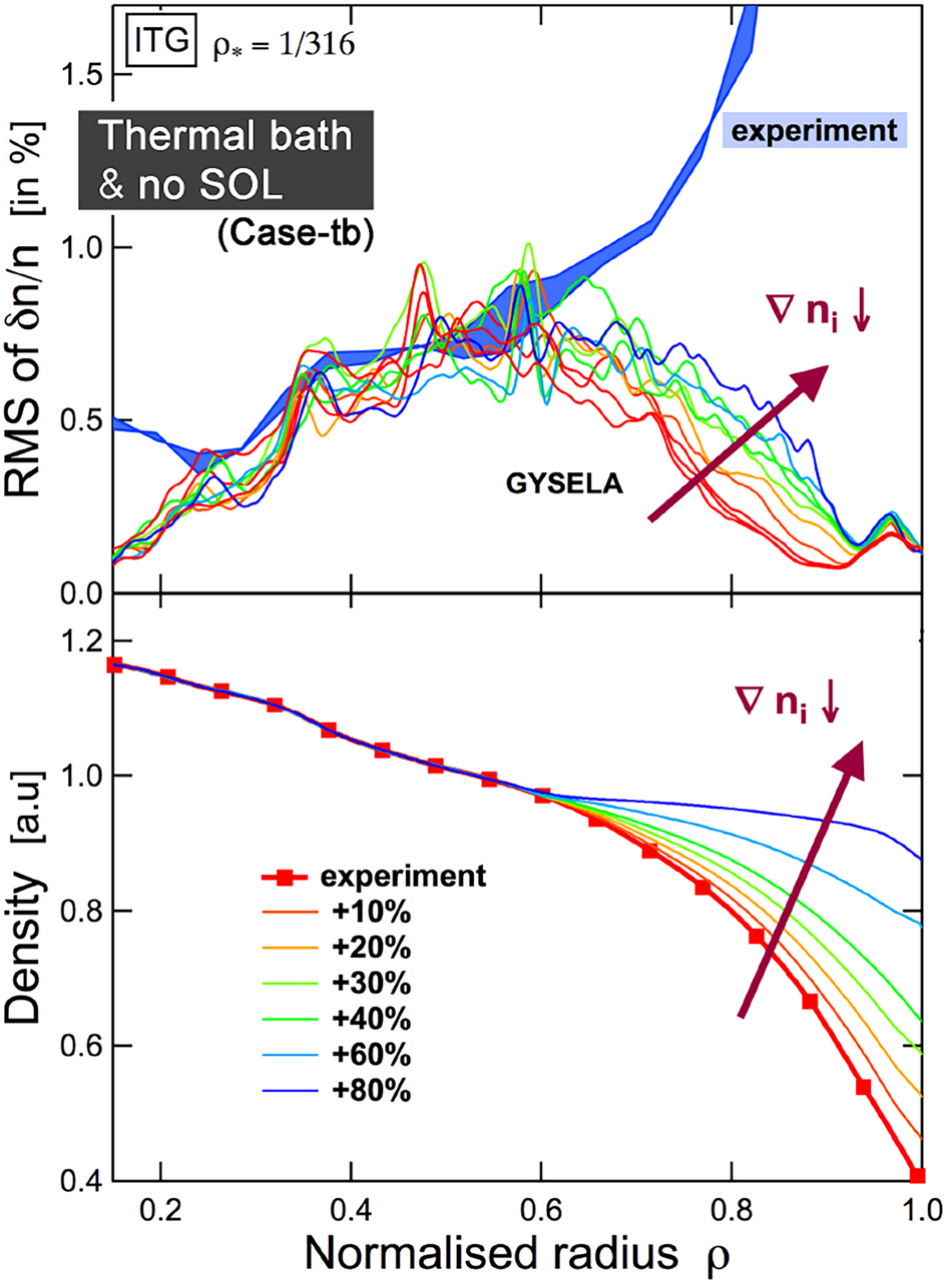
The upper panel illustrates the response of relative fluctuation levels $\delta n/n$ to incremental relaxation past $r/a\ge 0.6$ of density gradients (beyond experimental uncertainties), displayed in the bottom panel. The experimental measurements of $\delta n/n$ from fast-swept reflectometry (solid blue, top panel) demonstrate a consistent increase in these relative fluctuation levels, indicating a significant discrepancy between the computed and experimental values for $r/a\ge 0.65$

**Fig. 4:**
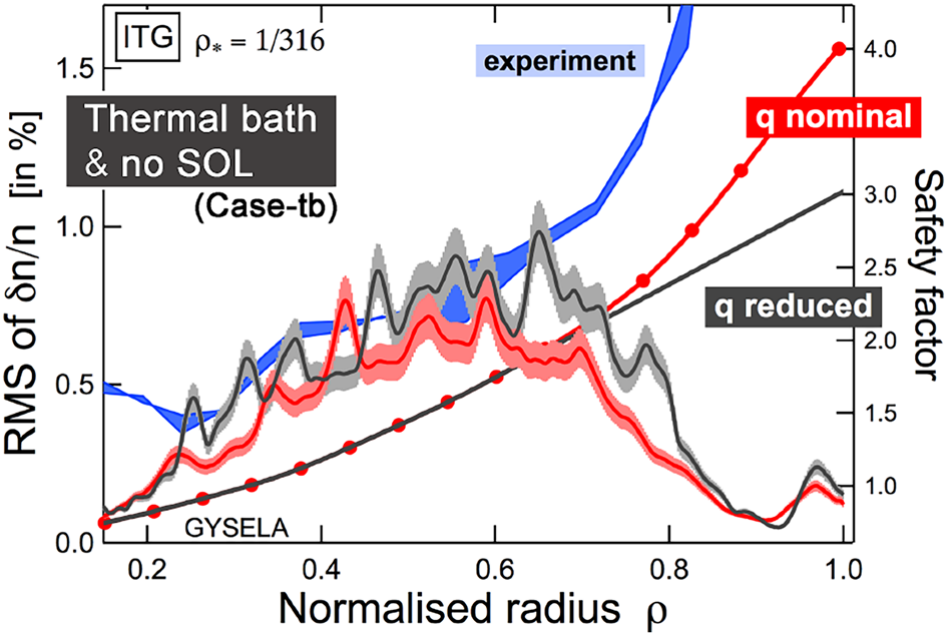
Modifying the safety factor past $r/a\ge 0.6$ (beyond experimental uncertainties) only modestly impacts fluctuations levels at the edge

We initialize gysela with profiles and parameters based on the Tore Supra shot 45511, as described above, and run a series of "thermal bath" computations. These calculations are, on purpose, quite large with $\rho_\star=1/316$ so as to be in the so-called ‘local limit’ (Lin et al. 2002), where local or gradient-driven approaches are often stated to be valid. The following questions this common understanding. In lieu of the penalized poloidally symmetric SOL of Case 2 where exponential relaxation to a cold Maxwellian is forced, here, an outer diffusive buffer region between $1\le r/a\le 1.3$ surrounds the confined plasma. In this ‘buffer’ region, the diffusion operator ${\mathcal {D}}$ (detailed above) ensures smooth damping of fluctuations that may have entered this peripheral buffer region. At experimental nominal values of the plasma profiles, a significant transport shortfall is consistently found, as illustrated in Figs. 3 and 4.

We now turn to an assessment of the sensitivity of edge transport dynamics to variations in input profiles, beyond experimental uncertainties, and an evaluation of the incidence of artificial diffusion on edge transport. Given the stabilizing effect of density gradients on the dominant Ion Temperature Gradient (ITG) instability within the specified parameter range, we proceed to examine their influence on the edge. In order to achieve this, the density profiles in Fig. 3 are maintained at their original values for $r/a \le 0.6$ and then gradually flattened beyond this point, up to $r/a =1$. As anticipated, the levels of ITG-driven turbulence in the edge increase although they remain significantly below the levels observed in the experiment. Subsequently, the sensitivity of the edge turbulence levels to the safety factor *q* and the magnetic shear *s* is then evaluated as shown in Fig. 4. For $r/a \ge 0.6$, the safety factor is reduced from $q_{95}=3.7$ (nominal case throughout the paper) to $q_{95}=3$. A decrease in turbulence intensity levels is expected (and has been studied in Ref. Varennes et al. (2023), in the limiter configuration). The variation in levels of turbulence intensity is modest, and uncertainties in the *q* profile are unlikely to cure the observed shortfall at the edge.

### Includes modeling of scrape-off layer (SOL)

**Fig. 5:**
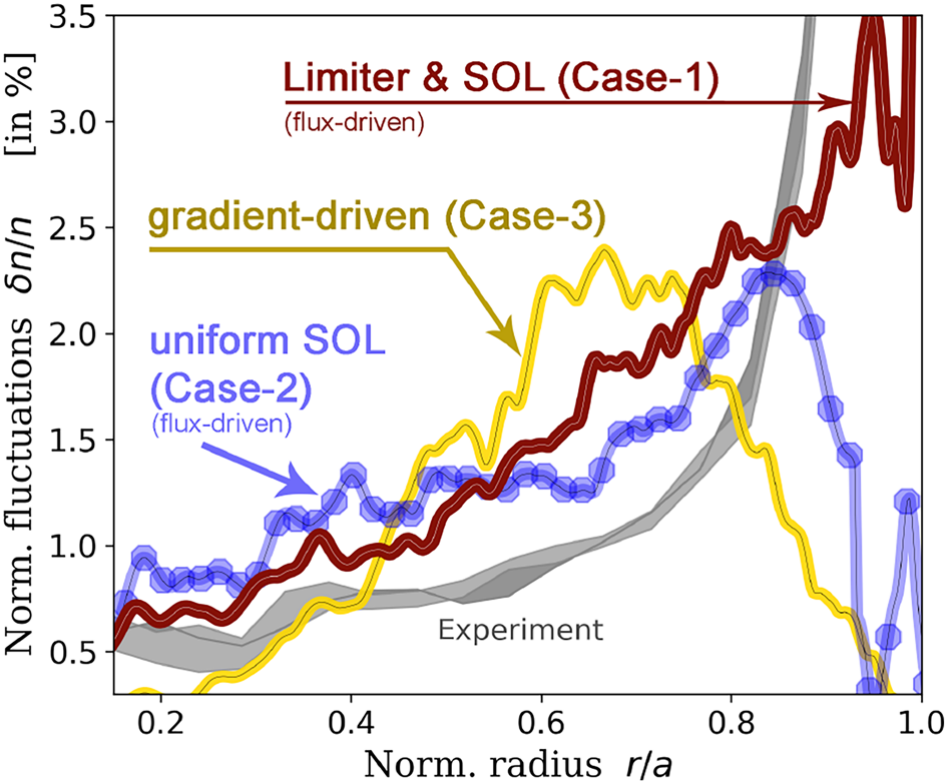
Differences in forcing and boundary conditions show clear differences in how turbulence globally organizes, at statistical equilibrium. This illustrates the central roles played by the spreading of turbulence and the magnetic connection to the material boundaries (or, more generally, the existence of a local cold spot). Adapted from Dif-Pradalier et al. (2022), Fig. 4

In order to gain a deeper understanding of the various physical processes at play, we proceed to include modeling of the scrape-off layer and evaluate the role of forcing and boundary conditions. Extensive tests have been performed to assess the robustness of the reported results in Ref. Dif-Pradalier et al. (2022). Gradient anisotropy and magnitudes of Cases 1, 2, and 3 are robust while varying distribution function initialization (local or canonical Maxwellians), presheath values in the SOL through $\Lambda$ scans (from 0 to 5, its nominal value for Deuterium being $\Lambda \sim 4.1$), target penalized temperature $T_{\mathrm{{w}}}$ in the limiter and wall, limiter shape (large and flat, narrow, rounded) and poloidal location (bottom, top). They are summarized in Fig. 5.

Cases 1 and 2 are initialized with the same parameters, based on the Tore Supra shot 45511 as described above. For each computation, $\delta n/n$ is averaged at flux equilibrium over $30\,000\,\Omega _{c,i}$—which is larger than a typical linear growth time—and plotted, respectively, in burgundy and blue. Here, $\Omega _{c,i}$ denotes the ion cyclotron frequency. As detailed in Ref. Gillot et al. (2023) to compare flux-driven with gradient-driven computations, Case 2 is run until statistical equilibrium and its resulting profiles are then both time-averaged at steady state over $30\,000\,\Omega _{c,i}$ and radially smoothed through sliding windows of $60\,\rho _i$ to smear out flux-driven specificities (corrugations, staircases,...). These smooth profiles, based on Case 2, are used as input for the gradient-driven Case 3. At statistical equilibrium, $\delta n/n$ is again averaged over $30\,000\,\Omega _{c,i}$ and displayed for all 3 cases, in Fig. 5. The experimental measurement from fast-swept reflectometry (Clairet et al. 2010) is further displayed in light gray.

Compared to a similar flux-driven approach, a gradient-driven framework will essentially over-damp zonal mean flows, minimize transport that may be ballistic in nature, and hinder phenomena such as turbulence spreading. These points are discussed in detail in Ref. Dif-Pradalier et al. (2025). For here, it is sufficient to say that:

(i) Comparison of Case 2 with Case 3 therefore provides a quantitative estimate of the importance of turbulence spreading.

**Fig. 6:**
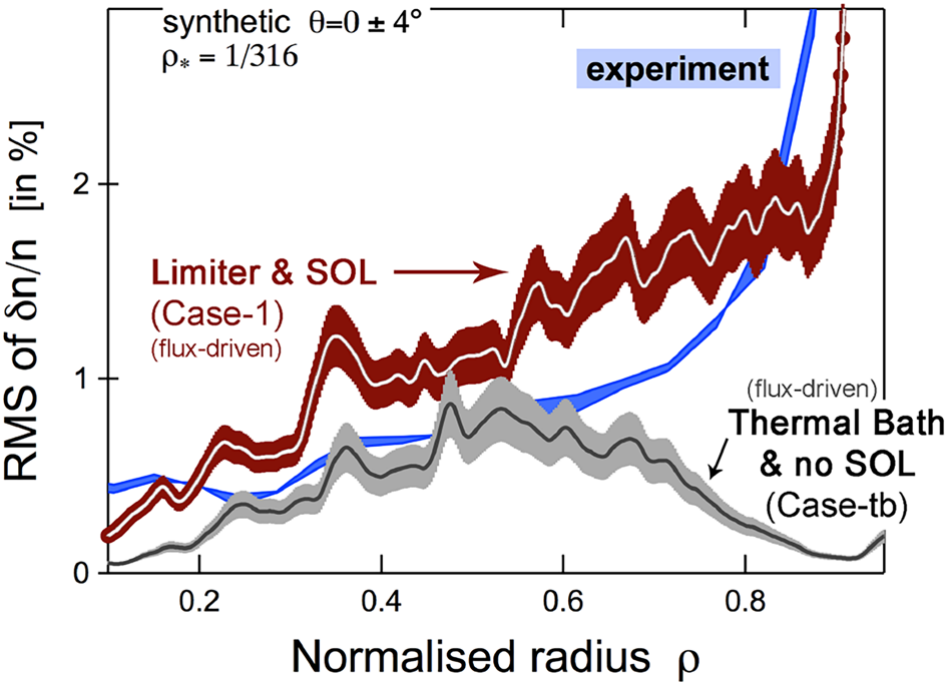
Role of boundary conditions and forcing on the calculated turbulence levels in the confined plasma (core and edge). The initial profiles are the same for both computations. Both are flux-driven and have the same capabilities in terms of turbulence spreading. Only the source to be spread differs. In the thermal bath approach, the system is weakly driven by coupling to external free energy reservoirs at both the inner and outer boundaries. This can be seen by comparing Case tb to Case 2 (Fig. 5), for the core plasma. More interestingly, Case tb (like Case 2) lacks the source of free energy from interaction with the boundaries that only Case 1 has via the limiter, resulting in a significant transport shortfall, i.e., an under-prediction of turbulent fluctuations at the edge ($\rho =r/a\ge 0.7$)

The interaction with the material boundaries or, more generally, the additional source of free energy for the edge resulting from the interaction with a cold spot is only present in Case 1 due to the limiter. Therefore,

(ii) Comparison of Case 2 with Case 1 provides a quantitative estimate of the role of this additional source in the global organization of the turbulence. Both cases are indeed flux-driven and have exactly the same spreading capabilities, but not the same source to be spread;
(iii) Comparison of Case 1 with Case tb (discussed in Figs. 3 and 4) additionally shown in Fig. 6 the importance of this additional source in the edge, resulting from the interplay with the material boundaries, but also shows the role of the boundary conditions: Case 1 and Case tb have exactly the same spreading capabilities outside the diffusive buffer regions, but not the same sources to be spread, both at the outer and the inner boundaries.

It is clear that both ingredients: (i) the spreading of patches of turbulent intensity far beyond their birth site and (ii) the interplay with a local cold spot, e.g., through a magnetic connection to the material boundaries, are central to recover the robust experimental trend of increasing $\delta n/n$ in the plasma edge.

As a final comment, while reducing the plasma column from a Tore Supra value of $\rho _\star = 1/500$ to a more numerically manageable $\rho _\star = 1/300$, the amount of power (the source numerically imparted to the plasma in a flux-driven context) should also have been reduced. However, since the $\rho _\star$ scaling of this power dependence is unclear, we have decided to still give the flux-driven calculations a (probably overestimated) equivalent of the 3 MW Tore supra experiment, which probably results in the $\delta n/n$ fluctuation levels in Cases 1 and 2 still being slightly high compared to the experiment.

### (Separatrix $\rightarrow$ core) and (core $\rightarrow$ separatrix) spreading

**Fig. 7:**
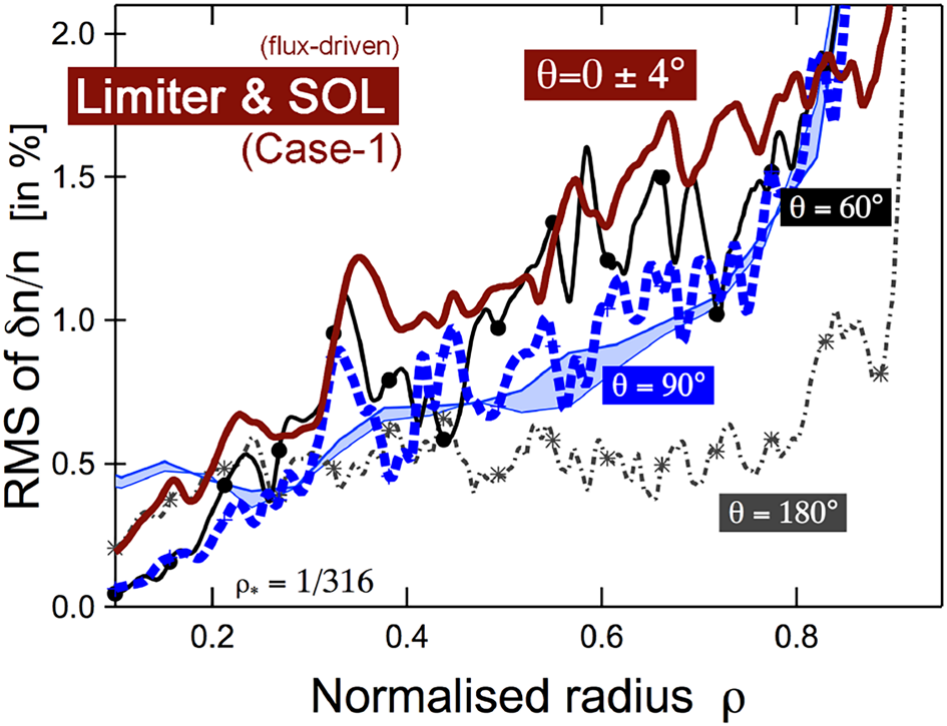
Poloidal distribution of turbulence levels in Case 1, at equilibrium. The low level of turbulence activity at the high-field side tends to indicate that a synergetic reinforcement of (separatrix $\rightarrow$ core) spreading of edge turbulence and (core $\rightarrow$ separatrix) spreading of core turbulence is likely required to sustain stable and large turbulence activity in steady-state

The resulting turbulence levels throughout the edge in Case 1 depend on the poloidal redistribution of the localized free energy source located around the limiter position at the poloidal angle $\theta =-90^\circ$. In the steady state, the (separatrix $\rightarrow$ core) propagation of the separatrix turbulence must equilibrate with the (core $\rightarrow$ separatrix) spreading of the core turbulence, which is ballooned due to the nature of the interchange instability. Figure 7 illustrates how these 2 trends combine to produce poloidally distributed turbulence levels.

Profiles are such that turbulence is marginally stable for $r/a\ge 0.84$ and linearly stable everywhere in the poloidal plane beyond $r/a\ge 0.9$ (see next section). In Case 1 alone, turbulent fluctuations initially grow around the location of the limiter, at $(r/a\approx 0.98,\theta \approx -90^\circ )$, and quickly spread poloidally across the low-field side region, with full equilibration in about $1\,$ms (Dif-Pradalier et al. 2022). Interestingly, the initially localized turbulence activity spreads over most of the poloidal plane, including the high-field side region. However, in the steady state, the high-field side turbulence activity dies out as shown by the $\delta n/n$ low levels, at $\theta =180^\circ$ in Fig. 7.

This emphasizes the importance of the (core $\rightarrow$ separatrix) propagation of core turbulence in maintaining high levels of turbulence activity throughout the edge despite its secondary role to the (separatrix $\rightarrow$ core) propagation of boundary-borne turbulence. While (core $\rightarrow$ separatrix) spreading alone is not sufficient to resolve the observed transport shortfall, its efficiency is certainly enhanced by the edge destabilization resulting from the (separatrix $\rightarrow$ core) propagation of limiter-borne turbulence. Indeed, the efficient propagation of a turbulent vortex is facilitated by a region of persistent or existing turbulence activity, rather than by a laminar or fully stable region.

The destabilization of the low-field side edge in steady state is further enhanced by the ability of the ballooning core turbulence to spill over and propagate efficiently there. Conversely, although the high-field side edge is also initially destabilized by the early redistribution of the limiter-borne turbulence, the absence of active high-field side core turbulence to spill over and replenish the turbulence there is a very plausible reason as to why it fades away at statistical equilibrium.

In summary, (separatrix $\rightarrow$ core) propagation appears to be the primary trigger or facilitating mechanism for destabilizing the otherwise stable edge. A pre-destabilized edge increases spillover or spreading efficiency for distant sources of turbulence activity. To sustain turbulent activity over longer timescales, it is essential that core turbulence be able to spill over into the edge.

## Linear stability analysis

In the previous section, we documented how turbulence organizes non-linearly at the plasma edge. We have highlighted the basic physical mechanisms by which a transport shortfall can be observed or avoided under different forcings, boundary conditions, scans of plasma parameters and modeling strategies: flux-versus-gradient-driven approaches, i.e., with or without the assumption of scale separation between mean background and fluctuations. The importance of the non-linear and non-local nature (Dif-Pradalier et al. 2010; Ida et al. 2015; Sanchez and Newman 2015; Hahm and Diamond 2018) of the organization described above and in Ref. Dif-Pradalier et al. (2022) certainly depends on the outer edge being either marginally unstable or linearly stable. We now proceed to quantify this.

**Fig. 8:**
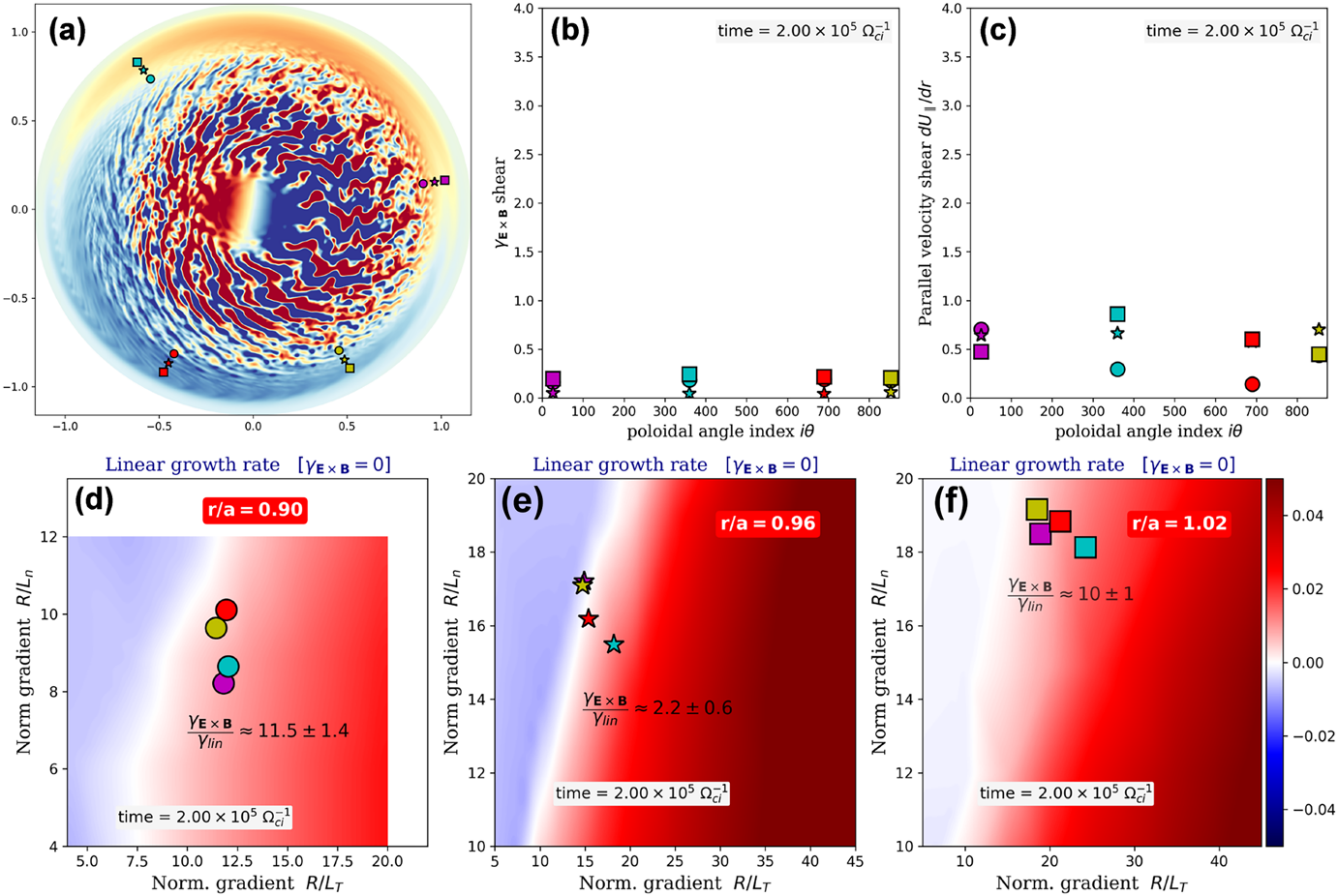
Data from flux-driven poloidally symmetric Case 2, at equilibrium ($t\Omega _{c,i}=200,000$). (a) Poloidal cross-sectional snapshot of the fluctuating electric potential. Specific locations are marked, combination of three radial locations: $\{r_1,r_2,r_3\}/a = \{0.90\text { (circles)}, 0.96\text { (stars)}, 1.02\text { (squares)}\}$ and four poloidal locations $\theta _k=\{9^{\circ }\text { (magenta)},126^{\circ }\text { (cyan)},-118^{\circ }\text { (red)},-61^{\circ }\text { (yellow)}\}$. Properties at these locations are shown in subplots (b): ${\textbf{E}}\times {\textbf{B}}$ shear, (c): parallel velocity shear and (d) through (f): maximal linear instability growth rate at vanishing ${\textbf{E}}\times {\textbf{B}}$ shear, keeping the same symbol–color combination

**Fig. 9:**
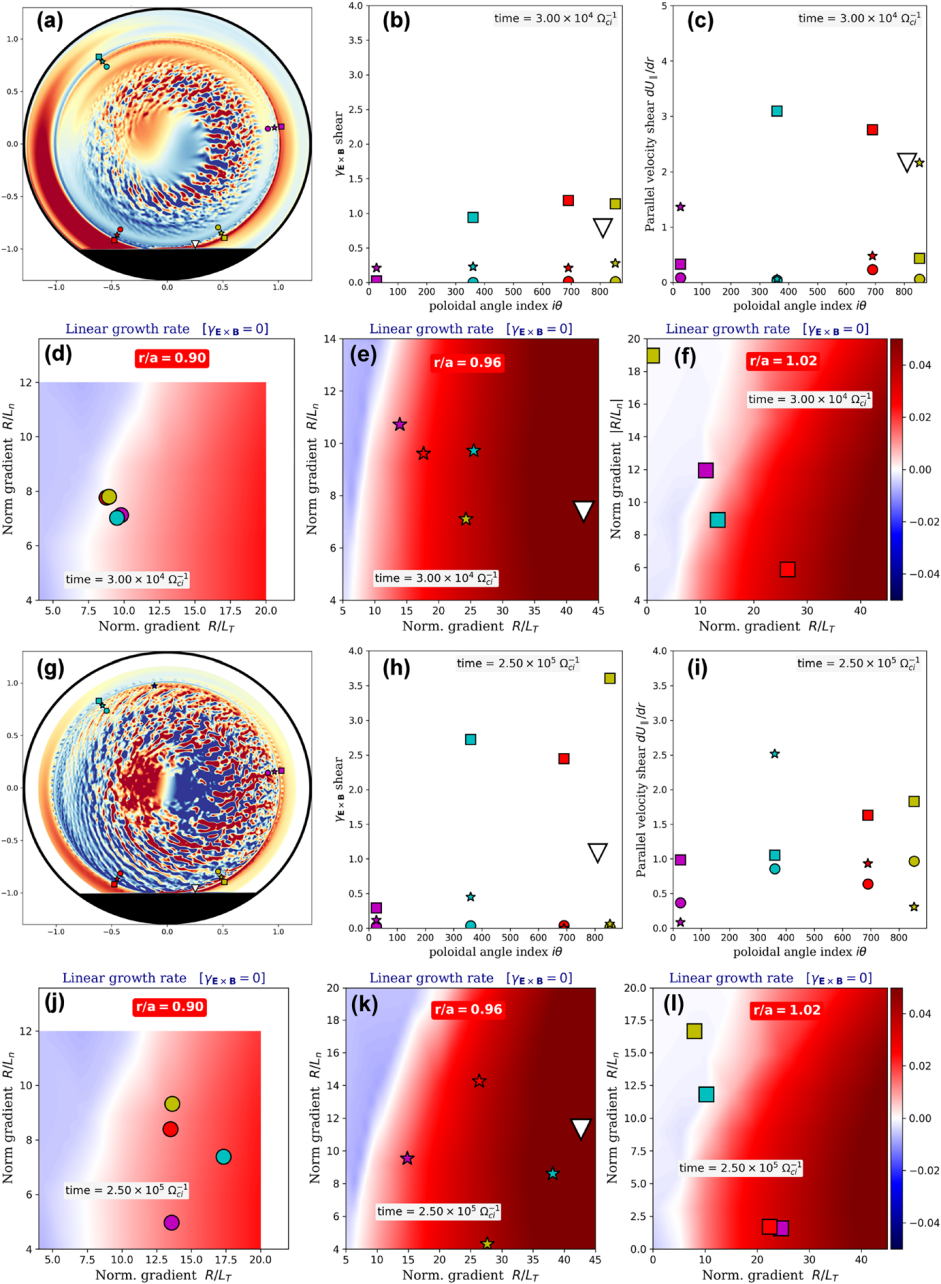
Same layout as in Fig. 8. Data are from flux-driven Case 1 with limiter at two different times: subplots (a) through (f) are early in the nonlinear development of turbulence ($t\Omega _{c,i}=30,000$); subplots (g) through (l) at thermal equilibrium ($t\Omega _{c,i}=250,000$). An additional location near the limiter at $(r/a=0.96,\theta =-75^{\circ })$, marked by the large white triangle is shown and corresponds to the region of maximum linear instability growth

To do so, a series of linear computations were performed using the initial value framework of the gradient-driven and local (flux tube) Gyrokinetic Workshop (gkw) code (Peeters et al. 2009). The inputs to gkw are all based on the output profiles of gysela at equilibrium, which in turn are based on those of the Tore Supra shot 45511 (see section 3). Two series of linear analyses are carried out. The first one (Fig. 8) is based on the equilibrium profiles of Cases 2 and 3, which are exactly the same by design. They study the dominant instabilities (interchange, parallel velocity gradient) in the absence of the limiter. The second series (Fig. 9) is similar, except for the fact that it is based on the equilibrium profiles of Case 1 and therefore includes the limiter and the magnetic connection to the material boundaries.

Unless stated otherwise (see Strategy III below), Boltzmann electrons have been assumed, as in gysela. Growth rates for the most unstable poloidal wavenumbers $k_\theta \rho _i = 0.6$ in the poloidally symmetric (Fig. 8) and limited (Fig. 9) configurations are estimated by the following procedure:

(i) at a given radial location $r_0$, compute the set $\Sigma _{r_0}=\{q, s, \nu _\star , T_i/T_e, U', \gamma _E, R/L_T, R/L_n\}$ of local values of the gysela plasma parameters, *q* being the safety factor, $s= (r/q)dq/dr$ the magnetic shear, $\nu _\star$ is the collisionality, $U'$ the parallel flow shear, $\gamma _E$ denotes the ${\textbf{E}}\times {\textbf{B}}$ shear, *R* the tokamak major radius and $L_X^{-1} = -d(Log X)/dr$ with $X=\{T,n\}$, the local logarithmic gradients of respectively ion temperature and density;
(ii) within gkw, the local approximation requires mean gradients to be constant over the computational domain since this is required by the numerical representation of a flux tube. The flux-driven gysela values are thus coarse-grained over a typical radial turbulence length scale $\Delta r=10 \rho _i$ to preserve the required information about the locality of the linear analysis. They are also averaged in time over the observed linear growth $\Delta t\,\Omega _{c,i}=25\,10^3$ of turbulent fluctuations. Furthermore, as the computational domain of gkw winds around the torus parsing both poloidal and toroidal angles, the background state is assumed to be uniform. This implies poloidal homogeneity along the flux tube and requires further averaging the gysela values on a flux surface $(\theta ,\varphi )$. In the end, all gysela observables in $\Sigma _{r_0}$ are averaged as follows: $\langle \langle \langle \langle \Sigma _{r_0} \rangle _{\Delta t} \rangle _{\Delta r}\rangle _\theta \rangle _\varphi$ before being used as input in gkw. Here, $\langle \cdot \rangle _\zeta$ denotes the average over $\zeta$. Physically, it amounts to estimating the instability drive as if located at its ballooning angle, effectively maximizing it;
(iii) for a chosen radial location $r_j$, knowing $\langle \langle \langle \langle \Sigma _{r_j} \rangle _{\Delta t} \rangle _{\Delta r}\rangle _\theta \rangle _\varphi$ allows to compute with gkw 2-dimensional maps of instability growth rates $\gamma _{lin}$ as a function of the logarithmic gradients (subplots Fig. 8d–f and Fig. 9d–f and j–l), each map tailored to the precise background local values in gysela for $T_i/T_e$, etc. Importantly, these maps are estimated in gkw at vanishing ${\textbf{E}}\times {\textbf{B}}$ shear, which, as in item (ii), further helps maximize the estimated linear growth rates. Neither the background ${\textbf{E}}\times {\textbf{B}}$ shear (the large-scale, static part often referred to as the ‘neoclassical’ part) nor its fluctuating component due to turbulence are included in gkw for linear calculations. The inclusion of such terms induces a shift in the radial wavenumbers (an advection in Fourier space), which results in the linear problem having no stationary solution. These terms are of course included in the non-linear calculations. The above is standard procedure in local "$\delta f$" approaches and differs from gysela because "full-f" calculations are always in force equilibrium, i.e., either during the linear growth or during the entire nonlinear evolution, the neoclassical equilibrium flow, the turbulence and their respective shears are always present;
(iv) we now estimate local values of the gysela local growth rates $\gamma _{\text {lin}}$ at 13 different radial–poloidal $(r_j,\theta _k)$ locations (shown in Fig. 1 as well), combination of three radial locations near the separatrix: $\{r_1,r_2,r_3\}/a = \{0.90\text { (circles)}, 0.96\text { (stars)}, 1.02\text { (squares)}\}$ and five poloidal locations spanning the entire poloidal cross section: $\theta _k=\{9^{\circ },126^{\circ },-118^{\circ },-75^{\circ },-61^{\circ }\}$;
(v) in order to assess actual stability, one can estimate an effective linear growth rate correcting for the ${\textbf{E}}\times {\textbf{B}}$ shear: $\gamma ^\text {eff} = \gamma _{\text {lin}} - \gamma _E$ [strategy I] or run gkw nonlinearly, including $\gamma _E$ from gysela [strategy II]. This latter strategy is significantly more demanding numerically and only a few cases have been investigated. Furthermore, 2 additional runs with gkw have been performed at $(r/a=0.84,\theta =9^\circ )$ and $(r/a=0.96,\theta =9^\circ )$ with a fully kinetic electron response to assess the impact of the Boltzmann electron approximation [strategy III].

Moving to the results:

- In all investigated analyses, the dominant instabilities inside $r/a \le 0.84$ are found to be of interchange character. With a Boltzmann electron response, the ion temperature gradient (ITG) is dominant. With a kinetic electron response the instability inside $r/a \le 0.84$ is a combination of ITG and Trapped electron modes (TEM);
- In poloidally symmetric Cases 2 and 3, gkw finds the edge to be marginally stable at vanishing ${\textbf{E}}\times {\textbf{B}}$ shear: $\gamma _{\text {lin}} \approx 0$ (Fig. 8d–f). When including ${\textbf{E}}\times {\textbf{B}}$ shear, strategy I predicts the edge to be nonlinearly unconditionally stable past $r/a\ge 0.9$, with $\gamma ^\text {eff} < 0$ for all radial–poloidal combinations considered. Strategies II and III confirm that location $(r/a=0.96,\theta =9^\circ )$ is indeed stable. Contributions of perpendicular and parallel shear flow (parallel velocity gradient, PVG) instability (D’Angelo 1965) are weak (Fig. 8c) and insufficient to destabilize the outer edge ($r/a\ge 0.9$), at any given poloidal location;
- In Case 1 with limiter the situation dramatically changes: destabilization of the outer edge starts in the vicinity of the limiter, just inside the separatrix. Figure 9e shows estimated ITG growth rates at $r/a=0.96$ that are larger than in Cases 2 and 3 while all locations at $r/a=0.9$ remain stable (Fig. 9a and d). Effective growth rates are large in the vicinity of the limiter at locations $\theta =-75^\circ$ and $-61^\circ$. Instability growth is also predicted at the plasma top $\theta =126^\circ$. Interestingly, as the limiter remains a cold sink throughout the nonlinear regime, the near-limiter drive endures (Fig. 9k). *This is a key result and certainly contributes to strengthen the conclusions of sections* 3.3*and* 3.2*regarding the importance of turbulence spreading and of the cold spot throughout the nonlinear evolution*. As turbulence spreads, formerly stable regions are destabilized (Fig. 9g and j);
- In Case 1, both ${\textbf{E}}\times {\textbf{B}}$ shear (Figs. 9b and h) and parallel velocity gradient (Figs. 9c and i) are significantly increased with respect to poloidally symmetric Cases 2 and 3. The significant velocity shear near the separatrix is found to contribute a modest albeit positive fraction of the global instability with $\gamma ^\text {PVG}\equiv |M_\parallel L_{v_\parallel }^{-1}| - | L_{n}^{-1} | \approx 0$, when averaged on a flux surface. Here $M_\parallel$ is the parallel Mach number. However, local values of parallel velocity shear on either side of the limiter can locally reach up to 3 to 5 times the mean, with $\gamma ^\text {PVG} \gtrsim 0$, which does not exclude the possibility of local excitation of parallel shear instability.

It is important to note that although this procedure provides information on the nature of the instabilities involved, two important and often made approximations in local approaches should be relaxed to accurately interpret the flux-driven dynamics near the separatrix:

A. First, the thumb rule $\gamma _{{\textbf{E}}\times {\textbf{B}}} / \gamma _{\text {lin}} \ge 1$ for mode stabilization. The local ${\textbf{E}}\times {\textbf{B}}$ shear rates obtained from the gysela calculations with the limiter are much larger than the shear rates computed in the poloidally symmetric cases without the limiter (compare Figs. 9b and h respectively with Fig. 8b). Strategy I, which balances maximum growth with ${\textbf{E}}\times {\textbf{B}}$ shear, would predict interchange modes to be stable in the outermost radial locations, especially around the limiter (consider, e.g., the white triangle locations in Fig. 9). Clearly, there is active turbulence growing so the thumb rule $\gamma _{{\textbf{E}}\times {\textbf{B}}} / \gamma _{\text {lin}} \ge 1$ for mode stabilization is just that...a thumb rule.
B. Second, the postulate of poloidal (parallel) homogeneity as flux tubes wind around the torus is reasonably accurate in the core, but clearly less justifiable for describing near-separatrix conditions in limiter configurations. Much of the non-linear destabilization of the linearly stable edge is a consequence of the onset of such poloidal inhomogeneities. In addition, additional stabilization mechanisms are possible, such as profile coupling or poloidal shift of the envelope mode. This linear analysis is therefore likely to provide an upper bound estimate of the true flux-driven instability growth. However, it has the merit of confirming that the boundary is linearly stable under poloidally symmetric boundary conditions and that it is locally destabilized in the vicinity of the cold sink, highlighting the central role of turbulence propagation in understanding the global equilibration of turbulence dynamics.

## Causal inference

Causality detection in information theory is actively discussed (Hlavackova-Schindler et al. 2007). The “Transfer Entropy” (*TE*) is one of many methods to address aspects of causal relationships between temporal time series. The aim of this section is to provide an accompanying pedagogical approach to the results discussed in Refs. Dif-Pradalier et al. (2022, 2021). *TE* is a simple nonlinear extension of Granger causality (Granger 1980), introduced by Schreiber (2000) and studied in magnetized plasmas by van Milligen et al. (2014). Complementary approaches, e.g., based on information geometry theory, have recently been discussed by Kim and Thiruthummal (2024) and certainly look promising to capture causal relationships between fluctuating flows, zonal mean flows and turbulence.

The idea behind *TE* is simple: let’s consider a time series $(x_i)$ of the observable *X*, with $0\le i \le n$. If one can better predict its next realization $x_{n+1}$ with additional data from another time series $(y_j)$ of the observable *Y* with $0\le j\le n$, then “Y transfers information (i.e., causes) X”, or more precisely as “Y forecasts X”, which constitutes the definition of causality here. This idea is quantified by measuring the deviation of the transition probabilities from independence, i.e., from a stationary Markov process. In its simplest expression, if processes *X* and *Y* are independent, then the following generalized Markov property holds for all $0\le k\le n$: $p(x_{n+1}|x_{n-k},y_{n-k}) = p(x_{n+1}|x_{n-k})$. The standard notation for conditional probabilities is used here: *p*(*a*|*b*) is the probability of *a* knowing *b*. If now processes *X* and *Y* are not independent, the ratio of these two transition probabilities gives a measure of how much information they can share. In other words, how much knowing values within *Y* in addition to past values in *X* can help better evaluate the next step $x_{n+1}$. This idea leads to the following definition of the Transfer Entropy (*TE*) from process *Y* to process *X*:

5 $$\begin{aligned} T\!E_{Y\rightarrow X}(k) = \sum p(x_{n+1}, x_{n-k}, y_{n-k}) \log \left( \frac{p(x_{n+1 } | x_{n-k}, y_{n-k}) }{ p(x_{n+1 } | x_{n-k}) } \right) \end{aligned}$$

where *k* is thus a time lag and represents the *k*-past of the time series *X* and *Y*. The summation process is detailed below, in Eq.(7). *TE* can equivalently be equivalently rewritten as a conditional mutual information and represents the additional amount of information that must be added to adequately represent the studied process $p(x_{n+1}|x_{n-k},y_{n-k})$ with respect to its reference Markov process $p(x_{n+1}|x_{n-k})$. In the absence of information flow from *Y* to *X*, the logarithm vanishes as state *Y* has no influence on the transition probabilities of *X*. It also follows that *TE* is directional, i.e., $TE_{Y\rightarrow X} \ne TE_{X \rightarrow Y}$, effectively allowing causality to be inferred between processes *X* and *Y*. *TE* has interesting properties: it is independent of the relative magnitudes of the signals *X* and *Y*, applies to both linear and nonlinear regimes, is easily evaluated in real (configuration) space rather than in Fourier space and is typically less statistically demanding than bispectral techniques. Practically, *TE* is evaluated by expressing the conditional probabilities as joint probabilities $p(x_{n+1}|x_{n-k}, y_{n-k}) = p(x_{n+1},x_{n-k},y_{n-k})/p(x_{n-k},y_{n-k})$ and computing the 4 multidimensional probability density functions (pdfs) in Eq.(6) as a function of time delay *k*. *TE* is then normalized so that $0\le TE\le 1$. The 4 pdfs in Eq.(6) result from a binning process of the time series *X* and *Y* and are estimated in practice as in Eq.(7):

6 $$\begin{aligned} T\!E_{Y\rightarrow X,\alpha }(k)&= \sum p^\alpha (x_{n+1}, x_{n-k}, y_{n-k}) \log ^\alpha \left( \frac{p(x_{n+1 }, x_{n-k}, y_{n-k}) \, p(x_{n-k}) }{ p(x_{n+1 }, x_{n-k}) \, p(x_{n-k}, y_{n-k})} \right) \end{aligned}$$

7 $$\begin{aligned} T\!E_{Y\rightarrow X,\alpha }(k)&= \sum _{i=1}^{\beta }\sum _{j=1}^{\beta }\sum _{l=1}^{\beta }\, \left[ p^{3d}(i,j,l)\right] ^\alpha \log ^\alpha \left( \frac{p^{3d}(i,j,l) \, p^{1d}(j) }{ p^{2d}_{xx}(i,j)\,p^{2d}_{xy}(j,l) } \right) \end{aligned}$$

where $p^{3d}$, $p^{2d}_{xx}$, $p^{2d}_{xy}$ and $p^{1d}$ are the discretized versions of $p(x_{n+1},x_{n-k},y_{n-k})$, $p(x_{n+1},x_{n-k})$, $p(x_{n-k},y_{n-k})$ and $p(x_{n-k})$, respectively. To obtain sufficient statistics, a bin size of $\beta =2$ or $\beta =3$ is typically chosen, depending on the available length of the time series (the longer the time series, the larger $\beta$ can theoretically be). We have introduced the additional exponent $\alpha \ge 1$, which effectively represents a nonlinear threshold: low probabilities are further reduced and higher ones are amplified. In Ref. Dif-Pradalier et al. (2022), the pdfs in Eq.(6) are discretized using $\beta ^d=2^d$ bins, where *d* is the dimensionality of the pdf. The nonlinear threshold exponent $\alpha$ is set to one and *X* and *Y* are discretized at the same rate and enter the *TE* calculation with zero temporal mean.

In a complex environment, information can flow both ways, from *Y* to *X* and inversely. It is therefore particularly useful to define the net transfer entropy $\Delta _{X,Y}(TE)[k] = TE_{Y\rightarrow X}[k] - TE_{X\rightarrow Y}[k]$, which gives the net information flow between processes *X* and *Y*, at time lag *k*.

### Manufactured solutions

**Fig. 10:**
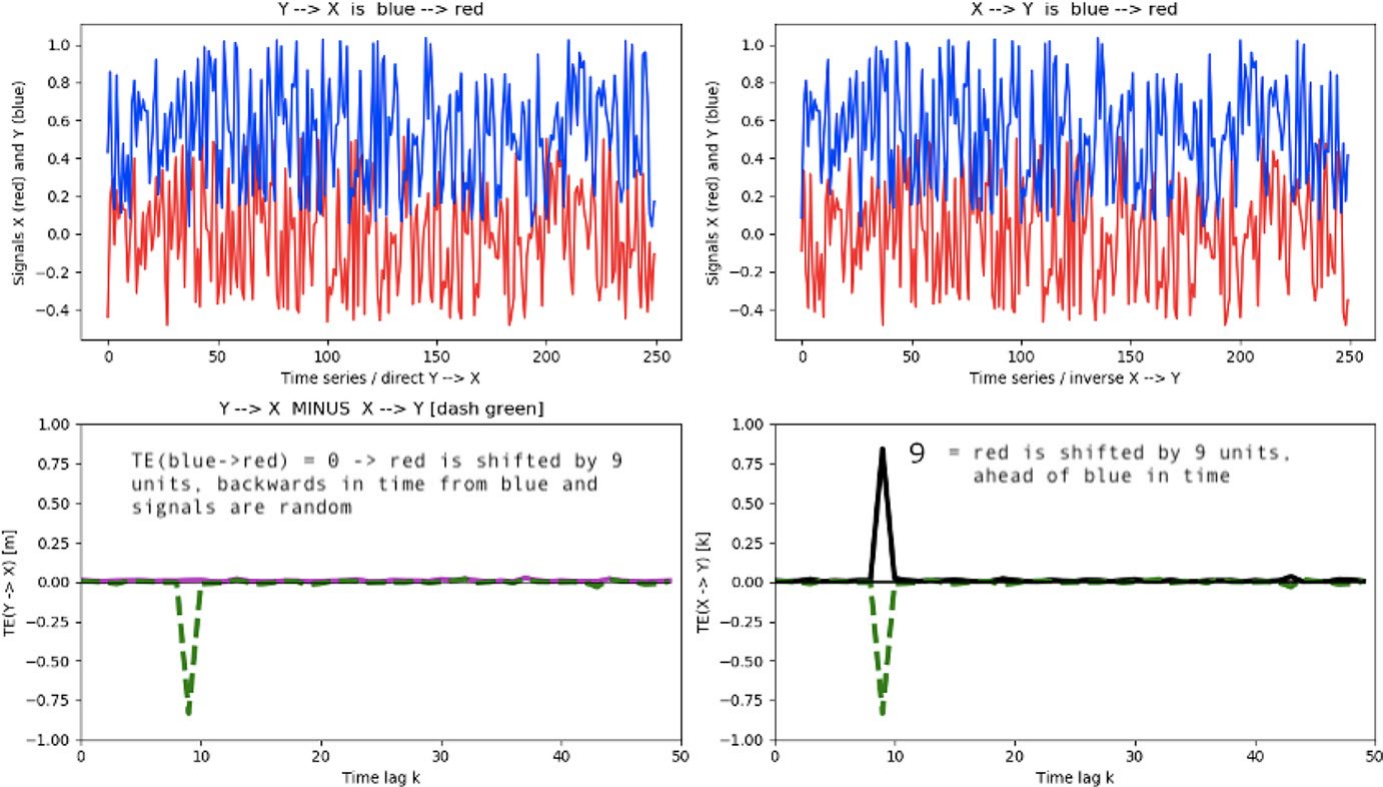
(Top left and right) Time traces of the two processes X and Y over which *TE* is computed. For visualization purposes only, Y is offset by its maximum value. $TE(Y\rightarrow X)$ is represented (lower left) in purple and $TE(X\rightarrow Y)$ in black (lower right) as a function of time lag k. The net Transfer Entropy $\Delta _{X,Y}(TE)$ is displayed as well (dashed green)

**Fig. 11:**
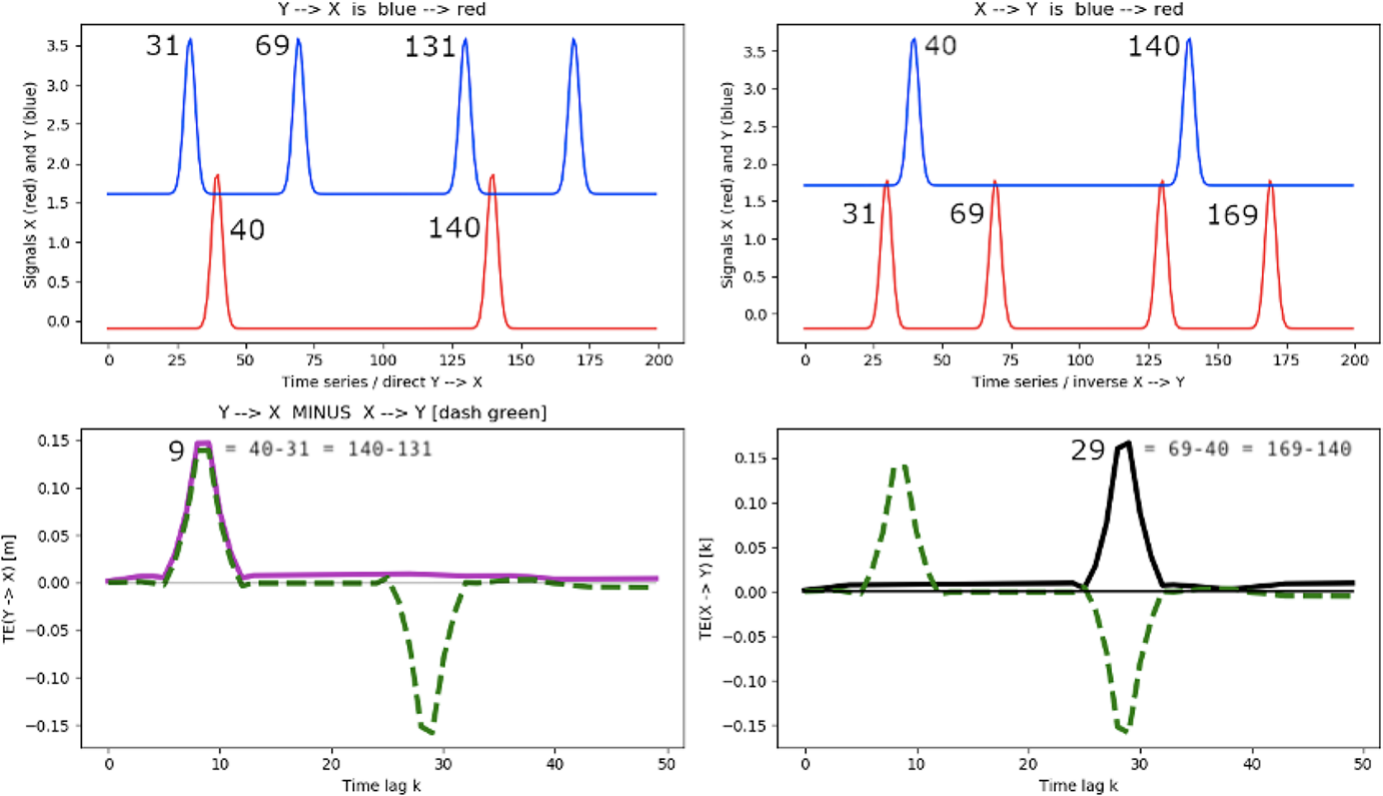
Time traces of processes X and Y consisting of a series of Lorentzian peaks and associated *TE* calculations; color coding and legends are as in Fig. 10

In order to provide a pedagogical introduction to the interpretation of transfer entropy graphs, let us detail the following manufactured solutions. In Fig. 10, we show the example for a random time series X. Y is constructed such that it exactly corresponds to X, with a forward in time delay of 9 units. As only past information may be shared between X and Y and as time lag k tests for similarities in shape of processes X and Y, $TE(Y\rightarrow X)$ should be vanishing for all time lags for a random time series and display a marked peak around time lag k = 9 for the $TE(X\rightarrow Y)$. The net TE simply states that for all the 50 time lags considered here, the only meaningful flow of information can go from $X\rightarrow Y$, with a time lag of 9.

In Fig. 11, we now consider two time series X and Y consisting of a series of Lorentzian peaks, again offset for visualization purposes only. Focusing on the left panel, the blue peak at $time=31$ from process Y can transfer information to the two peaks of process X at $time=40$ and 140, the former with a time lag of 9, the latter with a time lag of 109. Conversely, looking at the reverse $TE(X\rightarrow Y)$, the peak at $time=40$ can only transfer information to the peaks at $time=69$, 131, and 169 and not to the previous peak in $time=31$. This illustrates causality in the flow of time and clarifies the reason for the $TE(X\rightarrow Y)$ peaks at time lags of 29, 91 and so on (bottom right panel). It also shows that the TE technique does not require many occurrences of causal events to unambiguously extract relevant information flows.

**Fig. 12:**
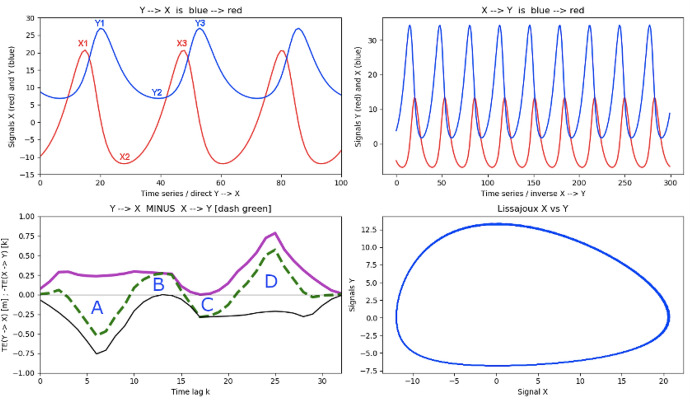
TE applied to the Lotka–Volterra equations; color coding and legends are as in Fig. 10

Another typical situation, common in nonlinear dynamics is illustrated in Fig. 12, which simulates predator–prey behaviour from the Lotka–Volterra equations:

8 $$\begin{aligned} \frac{du}{dt}&= au - buv \end{aligned}$$

9 $$\begin{aligned} \frac{dv}{dt}&= c u v -dv \end{aligned}$$

with parameters $a = 1,\ b = 0.1,\ c = 0.075$ and $d = 1.5$. The typical cyclical behaviour between predator and prey is readily apparent from the Lissajoux plot (bottom right panel) of process X (the prey) as a function of the predator Y. Where correlation analysis would merely pick up the periodicity of the predator and prey oscillations, this example illustrates the usefulness of the net Transfer Entropy $\Delta _{X,Y}(TE)$ (dashed green, lower left panel) in providing a guide to the flow of information, as the nonlinear system evolves. Individual TE curves provide information about typical waiting times for the system to reach a given state. For example, $TE(X\rightarrow Y)$ [black, bottom panel] tells us that, starting from a large number of prey (X1), the system takes about $k\sim 6$ time steps to reach a state with a large number of predators (Y1). Conversely, from $TE(Y\rightarrow X)$, starting from a state with many predators (Y1), the system will need $k\sim 25$ time steps to be able to support again a large number of prey (X3). Of course, in a complex system, information flows between prey and predator throughout the oscillation period. Net TE $\Delta _{X,Y}(TE)$ captures inflection points relevant to the dynamics. Thus, peak A corresponds to the situation where an increase in prey numbers or a large abundance of prey triggers a large increase in predators with a time lag of $\sim 6$. In stage B, predators in large numbers now eat more prey than their birth rate can compensate for, and their numbers are decimated. In stage C, fewer prey trigger a decline in predator numbers with a time lag of $\sim 18$. The low predator population in stage D now triggers an increase in prey growth with a time lag of $\sim 25$, and the system goes into a loop again.

### Practical application to the plasma edge

In practice, for example, one has time traces of a few significant observables, either from experiments or from calculations. An interesting, often non-trivial question to answer is "can we actually infer causal relationships between these observables? Inferring these causal relations is arguably a prerequisite for actually claiming to understand the underlying mechanisms, and is central to building reduced models that can capture these essential pieces of physics. Here we set out to test the basic mechanisms of flow shear enhancement at the plasma edge. We have therefore systematically applied this *TE* algorithm to actual time series from the flux-driven Case 1 calculation with limiter boundary conditions, in the last 5% inside the separatrix, where the spontaneous onset of a persistent transport barrier is observed. The above section, with its manufactured solutions, provides simple examples to interpret the *TE* technique and use the net transfer entropy $\Delta _{X,Y}(TE)$ as a ‘causality diagnostic’. Armed with a better understanding of the capabilities and typical outputs of the method, we can now aim to assess the causality of the underlying mechanisms responsible for flow shear growth. The details are highlighted in Ref. Dif-Pradalier et al. (2022) and we will only recall the main results below:

**Importance of diamagnetic stresses:** Vorticity balance clearly shows that both ${\textbf{E}}\times {\textbf{B}}$ and diamagnetic Reynolds stresses contribute to the generation of flow shear (Smolyakov et al. 2000; McDevitt et al. 2010; Ajay et al. 2020; Sarazin et al. 2021). This contribution of diamagnetics has long been known, but is often under-appreciated. Only recently has is been proven in computations that the magnitude of the diamagnetic Reynolds stress is in fact comparable to, if not consistently larger than, the ’normal’ electric Reynolds stress, both in the core (Sarazin et al. 2021) and at the edge (Dif-Pradalier et al. 2022);

**Dominant causal agent:** The relative weight of diamagnetic and electric Reynolds stresses in *magnitude* does not say much about how flows or barriers are *causally* generated. To this end, the *TE* causal analysis has revealed that diamagnetic flows ($v_{\star r}= -\partial _\theta p_\perp / r$ and $v_{\star \theta }= \partial _r p_\perp$) play the crucial role in the build-up phase of the flow shear at the edge, via diamagnetic advection of vorticity (i.e., diamagnetic Reynolds stresses). The ‘normal’ electric Reynolds stress, which is often expected to be the main driver of the flow, only becomes significant at later times, after the turbulent fluctuations have spread from the production region adjacent to the cold spot to a downstream region in the mid-plane. In the late stages of flow organization, diamagnetic and electric Reynolds stresses contribute roughly equally to vorticity production (i.e., to shear flow sustainment);

**Poloidal asymmetry, pressure inhomogeneity:** The central role of diamagnetic Reynolds stresses underlines the importance of poloidal asymmetries and pressure inhomogeneities in the nonlinear dynamics of turbulence;

**Possible implications for barrier formation:** Diamagnetic flows are driven by pressure inhomogeneities. It is argued here that Finite Larmor Radius (FLR) effects can be important for building up transport barriers. This implies that plasmas with similar global parameters may have different thresholds for accessing enhanced confinement states, depending on their vorticity production and vorticity content. These ideas in (Dif-Pradalier et al. 2022) are exploratory, based on electrostatic arguments, but independently echo earlier electromagnetic theories of transport bifurcations (Solano and Hazeltine Oct 2012) based on magnetic phase separation. This analogy deserves further investigation;

## Summarizing

Methods described throughout this paper and in Ref. Dif-Pradalier et al. (2022) have allowed to establish the following:

(i) The outer edge of the confined plasma can maintain experimentally consistent levels of turbulence despite being marginally unstable or even linearly stable to drift wave and interchange activity, due to the combined action of two ingredients. First, the interplay between the hot confined plasma and a cooler and localized region (a ‘cold spot’), which acts as an effective heat, particle or momentum sink. This cold spot can be created by magnetic coupling to the boundaries, as described here, or by a radiative X-point. This gives rise to a novel and local source of free energy in the vicinity of the cold spot. In other words, **the interaction between the plasma and its boundary profoundly modifies the convective stability near the magnetic separatrix.** This free energy is responsible for the onset of strong, albeit local, turbulent activity. Second, the possibility that these locally generated patches of turbulence propagate and destabilize distant regions of the edge and core. A globally organized state emerges in which the long-range propagation (‘spreading’) of these locally generated eddies, ‘non-locally’ (Dif-Pradalier et al. 2010; Ida et al. 2015; Sanchez et al. 2015; Hahm and Diamond 2018) controls the edge turbulence and the resulting organization;
(ii) Clearly, the *local* turbulence levels are not solely determined by the *local* values of key observables, such as the safety factor profile, or by the *local* values of the thermodynamic forces alone (the local gradients), as demonstrated by comparing Case tb with Case 1, Case 2 with Case 1 and Case 3 with Case 2. Linear analysis of the non-linear profiles in regions of sustained turbulence activity is found to be either marginally unstable or linearly stable. **Instead, the local turbulence levels in the edge are governed by boundary conditions and non-local fluxes of turbulence activity**;
(iii) **The absence of one or both of the above ingredients** (cold spot and spreading), either due to scale-separation assumptions (as in gradient-driven Case 3) or to inadequate boundary conditions (as in flux-driven Case 2 or Case tb) consistently leads to **transport shortfalls** of varying severity at the outer edge and **prevents the onset of a transport barrier** at the separatrix;
(iv) Following the initial importance of (separatrix $\rightarrow$ core) spreading of the turbulence activity in the vicinity of the cold spot, it seems highly plausible that a synergistic enhancement of both (separatrix $\rightarrow$ core) and (core $\rightarrow$ separatrix) spreading is required to maintain stable and large turbulence activity in the steady state;
(v) **Flow shear builds up** as eddies (vorticity) are advected, primarily by pressure inhomogeneities, i.e., **via diamagnetic Reynolds stresses**. The expanding turbulent interface, initially supported only in the vicinity of the cold spot, rapidly organizes into a stable peripheral transport barrier. Only at later times does the ‘normal’ electric Reynolds stress, which is often expected to be the main driver of the flow, become significant. In the steady state, diamagnetic and electric Reynolds stresses contribute approximately equally to shear flow sustainement.

## Data Availability

Raw data generated by the gysela code is available from the corresponding author upon reasonable request.

## References

[CR1] C.J. Ajay, S. Brunner, B. McMillan, J. Ball, J. Dominski, G. Merlo, How eigenmode self-interaction affects zonal flows and convergence of tokamak core turbulence with toroidal system size. J. Plasma. Phys. **86**(5), 905860504 (2020). 10.1017/S0022377820000999

[CR2] A.J. Brizard, T.S. Hahm, Foundations of nonlinear gyrokinetic theory. Rev. Mod. Phys. **79**(2), 421–468 (2007). 10.1103/RevModPhys.79.421

[CR3] E. Caschera, G. Dif-Pradalier, Ph. Ghendrih, V. Grandgirard, Y. Asahi, N. Bouzat, P. Donnel, X. Garbet, G. Latu, C. Passeron, Y. Sarazin, Immersed boundary conditions in global, flux-driven, gyrokinetic simulations. J. Phys. Conf. Ser. **1125**, 012006 (2018). 10.1088/1742-6596/1125/1/012006

[CR4] F. Clairet, S. Heuraux, C. Bottereau, D. Molina, L. Ducobu, F. Leroux, A. Barbuti, Fast sweeping reflectometry upgrade on tore supra. Rev. Sci. Instrum. **81**(10), 10D903 (2010). 10.1063/1.3460624 10.1063/1.3460624 21033935

[CR5] N. D’Angelo, Kelvin–helmholtz instability in a fully ionized plasma in a magnetic field. Phys. Fluid. **8**(9), 1748–1750 (1965). 10.1063/1.1761496

[CR6] P.H. Diamond, T.S. Hahm, On the dynamics of turbulent transport near marginal stability. Phys. Plasmas. **2**(10), 3640–3649 (1995). 10.1063/1.871063

[CR7] G. Dif-Pradalier, Ph. Ghendrih, Y. Sarazin, X. Garbet, V. Grandgirard, Y. Munschy, R. Varennes, L. Vermare, Y. Camenen and F. Widmer. Interplay between core, edge and scrape-off layer in Turbulent magnetised plasmas. *FEC 2020 - IAEA Fusion Energy Conference, May 2021, E-Conference, France. Preprints at* https://nucleus.iaea.org/sites/fusionportal/Shared%20Documents/FEC%202020/fec2020-preprints/preprint0888.pdf and https://cea.hal.science/cea-03287143v1 (2021)

[CR8] G. Dif-Pradalier, Y. Sarazin, D.W. Hughes, et al. Forcing and scale separation: what it entails for turbulence organisation. *Preparation*, (2025)

[CR9] G. Dif-Pradalier, P.H. Diamond, V. Grandgirard, Y. Sarazin, J. Abiteboul, X. Garbet, Ph. Ghendrih, A. Strugarek, S. Ku, C.S. Chang, On the validity of the local diffusive paradigm in turbulent plasma transport. Phys. Rev. E **82**(2), 025401(R) (2010). 10.1103/PhysRevE.82.025401 10.1103/PhysRevE.82.025401 20866867

[CR10] G. Dif-Pradalier, G. Hornung, X. Garbet, Ph. Ghendrih, V. Grandgirard, G. Latu, Y. Sarazin, The staircase of magnetised plasmas. Nucl. Fus. **57**(6), 066026 (2017)

[CR11] G. Dif-Pradalier, P. Ghendrih, Y. Sarazin, E. Caschera, F. Clairet, Y. Camenen, P. Donnel, X. Garbet, V. Grandgirard, Y. Munschy, L. Vermare, F. Widmer, Transport barrier onset and edge turbulence shortfall in fusion plasmas. Commun. Phys. **5**(1), 229 (2022). 10.1038/s42005-022-01004-z

[CR12] P. Donnel, K. Obrejan, Y. Sarazin, R. Bigué, E. Bourne, L. De Gianni, G. Dif-Pradalier, X. Garbet, V. Grandgirard, P. Krah, Y. Munschy, and M. Protais. An adaptative quasi-neutrality solver for full-f flux-driven gyrokinetic simulations of tokamak plasmas in presence of poloidal asymmetries. *Preparation*, (2025)

[CR13] X. Garbet, L. Laurent, A. Samain, J. Chinardet, Radial propagation of turbulence in tokamaks. Nucl. Fus. **34**(7), 963–74 (1994)

[CR14] C. Gillot. Model reduction for tokamak plasma turbulence: beyond fluid and quasi-linear descriptions. *PhD thesis, Aix-Marseille University*, (2020)

[CR15] C. Gillot, G. Dif-Pradalier, Y. Sarazin, C. Bourdelle, A. Bañón Navarro, Y. Camenen, J. Citrin, A. Di Siena, X. Garbet, V. Ph Ghendrih, P Manas Grandgirard, F. Widmer, The problem of capturing marginality in model reductions of turbulence. Plasma Phys. Controll. Fus. **65**(5), 055012 (2023). 10.1088/1361-6587/acc276

[CR16] T. Görler, A.E. White, D. Told, F. Jenko, C. Holland, T.L. Rhodes, A flux-matched gyrokinetic analysis of diii-d l-mode turbulence. Phys. Plasmas. **21**(12), 122307 (2014). 10.1063/1.4904301

[CR17] V. Grandgirard, J. Abiteboul, J. Bigot, T. Cartier-Michaud, N. Crouseilles, G. Dif-Pradalier, Ch. Ehrlacher, D. Esteve, X. Garbet, Ph. Ghendrih, G. Latu, M. Mehrenberger, C. Norscini, Ch. Passeron, F. Rozar, Y. Sarazin, E. Sonnendrücker, A. Strugarek, D. Zarzoso, A 5d gyrokinetic full- global semi-lagrangian code for flux-driven ion turbulence simulations. Comput. Phys. Commun. **207**, 35–68 (2016). 10.1016/j.cpc.2016.05.007

[CR18] C.W.J. Granger, Testing for causality: A personal viewpoint. J. Econ. Dyn. Control. **2**, 329–352 (1980). 10.1016/0165-1889(80)90069-X

[CR19] Ö.D. Gürcan, L. Vermare, P. Hennequin, V. Berionni, P.H. Diamond, G. Dif-Pradalier, X. Garbet, P. Ghendrih, V. Grandgirard, C.J. McDevitt, P. Morel, Y. Sarazin, A. Storelli, C. Bourdelle, The Tore Supra Team, Structure of nonlocality of plasma turbulence. Nucl. Fus. **53**(7), 073029 (2013)

[CR20] T.S. Hahm, P.H. Diamond, Mesoscopic transport events and the breakdown of fick’s law for turbulent fluxes. J. Korean. Phys. Soc. **73**(6), 747–792 (2018). 10.3938/jkps.73.747

[CR21] T.S. Hahm, P.H. Diamond, Z. Lin, K. Itoh, S.-I. Itoh, Turbulence spreading into the linearly stable zone and transport scaling. Plasma. Phys. Controll. Fus. **46**(5A), A323 (2004)

[CR22] K. Hlavackova-Schindler, M. Palus, M. Vejmelka, J. Bhattacharya, Causality detection based on information-theoretic approaches in time series analysis. Phys. Rep. **441**(1), 1–46 (2007). 10.1016/j.physrep.2006.12.004

[CR23] K. Ida, Z. Shi, H.J. Sun, S. Inagaki, K. Kamiya, J.E. Rice, N. Tamura, P.H. Diamond, G. Dif-Pradalier, X.L. Zou, K. Itoh, S. Sugita, O.D. Gürcan, T. Estrada, C. Hidalgo, T.S. Hahm, A. Field, X.T. Ding, Y. Sakamoto, S. Oldenbürger, M. Yoshinuma, T. Kobayashi, M. Jiang, S.H. Hahn, Y.M. Jeon, S.H. Hong, Y. Kosuga, J. Dong, S.-I. Itoh, Towards an emerging understanding of non-locality phenomena and non-local transport. Nucl. Fus. **55**(1), 013022 (2015)

[CR24] Y. Idomura, Accuracy of momentum transport calculations in full-f gyrokinetic simulations. Comput. Sci. Discover. **5**(1), 014018 (2012). 10.1088/1749-4699/5/1/014018/pdf

[CR25] B.B. Kadomtsev, E.W. Laing, *Tokamak Plasma: A Complex Physical System* (CRC Press, 1993)

[CR26] E.-J. Kim, A.A. Thiruthummal, Nonperturbative theory of the low-to-high confinement transition through stochastic simulations and information geometry: correlation and causal analyses. Phys. Rev. E **110**, 045209 (2024). 10.1103/PhysRevE.110.045209 39562906 10.1103/PhysRevE.110.045209

[CR27] P.C. Liewer, Measurements of microturbulence in tokamaks and comparisons with theories of turbulence and anomalous transport. Nucl. Fus. **25**(5), 543 (1985)

[CR28] Z. Lin, S. Ethier, T.S. Hahm, W.M. Tang, Size scaling of turbulent transport in magnetically confined plasmas. Phys. Rev. Lett. **88**(19), 195004 (2002). 10.1103/PhysRevLett.88.195004 12005641 10.1103/PhysRevLett.88.195004

[CR29] N. Mattor, P.H. Diamond, Drift wave propagation as a source of plasma edge turbulence. Phys. Rev. Lett. **72**, 486–489 (1994). 10.1103/PhysRevLett.72.486 10056445 10.1103/PhysRevLett.72.486

[CR30] C.J. McDevitt, P.H. Diamond, O.D. Gurcan, T.S. Hahm, Poloidal rotation and its relation to the potential vorticity flux. Phys. Plasmas. **17**(11), 112509 (2010). 10.1063/1.3490253

[CR31] B.F. McMillan, S. Jolliet, T.M. Tran, L. Villard, A. Bottino, P. Angelino, Long global gyrokinetic simulations: Source terms and particle noise control. Phys. Plasmas. **15**(5), 052308 (2008). 10.1063/1.2921792

[CR32] A. Mishchenko, A. Bottino, R. Hatzky, E. Sonnendrücker, R. Kleiber, A. Könies, Mitigation of the cancellation problem in the gyrokinetic particle-in-cell simulations of global electromagnetic modes. Phys. Plasmas. **24**(8), 081206 (2017). 10.1063/1.4997540

[CR33] A. Paredes, H. Bufferand, G. Ciraolo, F. Schwander, E. Serre, P. Ghendrih, P. Tamain, A penalization technique to model plasma facing components in a tokamak with temperature variations. J. Comput. Phys. **274**, 283–298 (2014). 10.1016/j.jcp.2014.05.025

[CR34] A.G. Peeters, Y. Camenen, F.J. Casson, W.A. Hornsby, A.P. Snodin, D. Strintzi, G. Szepesi, The nonlinear gyro-kinetic flux tube code gkw. Comput. Phys. Commun. **180**(12), 2650–2672 (2009). 10.1016/j.cpc.2009.07.001

[CR35] A.G. Peeters, F. Rath, R. Buchholz, Y. Camenen, J. Candy, F.J. Casson, S.R. Grosshauser, W.A. Hornsby, D. Strintzi, A. Weikl, Gradient-driven flux-tube simulations of ion temperature gradient turbulence close to the non-linear threshold. Phys. Plasmas. **23**(8), 082517 (2016). 10.1063/1.4961231

[CR36] R. Sanchez, D.E. Newman, Self-organized criticality and the dynamics of near-marginal turbulent transport in magnetically confined fusion plasmas. Plasma. Phys. Controll. Fus. **57**(12), 123002 (2015)

[CR37] Y. Sarazin, V. Grandgirard, J. Abiteboul, S. Allfrey, X. Garbet, Ph. Ghendrih, G. Latu, A. Strugarek, G. Dif-Pradalier, P.H. Diamond, S. Ku, C.S. Chang, B.F. McMillan, T.M. Tran, L. Villard, S. Jolliet, A. Bottino, P. Angelino, Predictions on heat transport and plasma rotation from global gyrokinetic simulations. Nucl. Fus. **51**(10), 103023 (2011)

[CR38] Y. Sarazin, G. Dif-Pradalier, X. Garbet, P. Ghendrih, A. Berger, C. Gillot, V. Grandgirard, K. Obrejan, R. Varennes, L. Vermare, T. Cartier-Michaud, Key impact of phase dynamics and diamagnetic drive on reynolds stress in magnetic fusion plasmas. Plasma. Phys. Controll. Fus. **63**(6), 064007 (2021). 10.1088/1361-6587/abf673

[CR39] O. Sauter, S. Brunner, D. Kim, G. Merlo, R. Behn, Y. Camenen, S. Coda, B.P. Duval, L. Federspiel, T.P. Goodman, A. Karpushov, A. Merle, TCV Team, On the non-stiffness of edge transport in l-mode tokamak plasmas. Phys. Plasmas. **21**(5), 055906 (2024). 10.1063/1.4876612

[CR40] T. Schreiber, Measuring information transfer. Phys. Rev. Lett. **85**, 461–464 (2000). 10.1103/PhysRevLett.85.461 10991308 10.1103/PhysRevLett.85.461

[CR41] A.I. Smolyakov, P.H. Diamond, M.V. Medvedev, Role of ion diamagnetic effects in the generation of large scale flows in toroidal ion temperature gradient mode turbulence. Phys. Plasmas. **7**(10), 3987–3992 (2000). 10.1063/1.1289514

[CR42] E.R. Solano, R.D. Hazeltine, Magnetic phase transitions in plasmas and transport barriers. Nucl. Fus. **52**(11), 114017 (2012). 10.1088/0029-5515/52/11/114017

[CR43] BPh. van Milligen, G. Birkenmeier, M. Ramisch, T. Estrada, C. Hidalgo, A. Alonso, Causality detection and turbulence in fusion plasmas. Nucl. Fus. **54**(2), 023011 (2014). 10.1088/0029-5515/54/2/023011

[CR44] R. Varennes, L. Vermare, X. Garbet, P. Hennequin, G. Dif-Pradalier, Y. Sarazin, V. Grandgirard, O. Panico, P. Donnel, K. Obrejan, Safety factor influence on the edge exb velocity establishment in tokamak plasmas. Plasma. Phys. Controll. Fus. **66**(2), 025003 (2023)

